# Open Surgical Management of Renal Cell Carcinoma with Infradiaphragmatic Venous Tumor Thrombus (Mayo Levels 0–III): The Epitome of Surgical Self-Reliance in Urology

**DOI:** 10.3390/cancers18071080

**Published:** 2026-03-26

**Authors:** Dorin Novacescu, Adelina Baloi, Silviu Latcu, Flavia Zara, Dorel Sandesc, Cristina-Stefania Dumitru, Cristian Condoiu, Razvan Bardan, Vlad Dema, Radu Caprariu, Talida Georgiana Cut, Alin Cumpanas

**Affiliations:** 1 Department II of Microscopic Morphology, Victor Babes University of Medicine and Pharmacy Timisoara, E. Murgu Square, No. 2, 300041 Timisoara, Romania; novacescu.dorin@umft.ro (D.N.); flavia.zara@umft.ro (F.Z.); cristina-stefania.dumitru@umft.ro (C.-S.D.); 2 Doctoral School, Victor Babes University of Medicine and Pharmacy Timisoara, E. Murgu Square, Nr. 2, 300041 Timisoara, Romania; adelina.baloi@umft.ro (A.B.); vlad.dema@umft.ro (V.D.); 3 Department X—Surgery II, Discipline Anesthesia and Intensive Care, Victor Babes University of Medicine and Pharmacy Timisoara, E. Murgu Square, Nr. 2, 300041 Timisoara, Romania; sandesc.dorel@umft.ro; 4 Department XV, Discipline of Urology, Victor Babes University of Medicine and Pharmacy Timisoara, E. Murgu Square, No. 2, 300041 Timisoara, Romania; razvan.bardan@umft.ro (R.B.); cumpanas.alin@umft.ro (A.C.); 5 Department XV, Discipline of Radiology and Medical Imaging, Victor Babes University of Medicine and Pharmacy Timisoara, E. Murgu Square, Nr. 2, 300041 Timisoara, Romania; radu.caprariu@umft.ro; 6 Department XIII, Discipline of Infectious Diseases, Victor Babes University of Medicine and Pharmacy Timisoara, E. Murgu Square, Nr. 2, 300041 Timisoara, Romania; talida.cut@umft.ro

**Keywords:** renal cell carcinoma, venous tumor thrombus, inferior vena cava, radical nephrectomy, thrombectomy, liver mobilization, open surgery

## Abstract

**Background/Objectives**: Renal cell carcinoma (RCC) with venous tumor thrombus (VTT) extending into the inferior vena cava (IVC) occurs in 4–10% of patients and represents one of the most technically demanding scenarios in urologic surgery. Open radical nephrectomy with en bloc thrombectomy remains the gold standard for infradiaphragmatic disease (Mayo Levels 0–III), offering the only realistic prospect for long-term cure. This narrative review provides a technically oriented, evidence-based guide for surgical urologists managing these complex cases. **Methods**: PubMed/MEDLINE, Scopus, and Web of Science were searched (1970–March 2025) using terms related to RCC, venous tumor thrombus, IVC thrombectomy, and perioperative management. Priority was given to prospective studies, systematic reviews, large retrospective cohorts, and current guidelines (EAU 2025, NCCN v2.2024). Original intraoperative photographs supplement procedural descriptions. **Results**: We detail the complete perioperative pathway: VTT classification (Mayo/AJCC), multimodal imaging, patient optimization, and level-specific open surgical techniques—ranging from Satinsky clamping for Level 0–I thrombi to full piggyback liver mobilization with hepatic vascular exclusion for Level III disease. Contemporary perioperative mortality is <2% at high-volume centers (reported in single and multicenter retrospective series from high-volume institutions), with 5-year cancer-specific survival of approximately 50–60% in non-metastatic cases. Adjuvant pembrolizumab is now a standard of care following the KEYNOTE-564 trial. Neoadjuvant immune checkpoint inhibitor plus tyrosine kinase inhibitor combinations show promising VTT downstaging rates (44–100%), though their role remains investigational. Robotic-assisted thrombectomy demonstrates favorable perioperative outcomes for Level I–II thrombi at experienced centers. **Conclusions**: Open surgery remains the cornerstone of curative treatment for RCC with infradiaphragmatic VTT, requiring meticulous preoperative planning and multidisciplinary collaboration at high-volume centers. Integration of perioperative systemic therapies and robotic-assisted approaches holds promise for further improving outcomes in this challenging patient population.

## Simple Summary

Renal cancer can extend beyond the kidney into the inferior vena cava—the body’s largest vein—as a venous tumor thrombus, occurring in approximately 4–10% of patients. Without surgical intervention, median survival is approximately five months. However, complete surgical removal of the kidney and the tumor thrombus in a single operation can achieve five-year cancer-specific survival rates exceeding 50% in patients without distant metastases. This surgery is among the most technically demanding in urology, requiring detailed preoperative imaging, multidisciplinary team coordination, and techniques adapted from liver transplantation surgery. This review provides a comprehensive, evidence-based guide covering preoperative evaluation, thrombus classification, patient optimization, and level-specific open surgical techniques for infradiaphragmatic disease (Mayo Levels 0–III). We also review the role of neoadjuvant and adjuvant systemic therapies, including immune checkpoint inhibitors, and the emerging application of robotic-assisted approaches for selected cases. The aim is to serve as a practical surgical reference for optimizing outcomes in this challenging patient population.

## 1. Introduction

Renal cell carcinoma (RCC) is the third most common genitourinary malignancy and is uniquely prone to vascular invasion [1,2]. RCC encompasses a heterogeneous group of malignancies arising from the renal tubular epithelium, with distinct histological subtypes that carry differing biological behaviors and clinical outcomes. Clear cell RCC (ccRCC) is the most prevalent subtype, accounting for approximately 70–80% of cases, and is characterized by loss of the von Hippel–Lindau (VHL) tumor suppressor gene on chromosome 3p, leading to upregulation of hypoxia-inducible factor (HIF) and downstream vascular endothelial growth factor (VEGF) signaling—a pathway central to both its highly vascular phenotype and its propensity for venous invasion [1]. Papillary RCC (types 1 and 2) constitutes 10–15% of cases, while chromophobe RCC accounts for approximately 5%. Rarer variants, including collecting duct carcinoma and unclassified RCC, comprise the remainder [1,2]. Sarcomatoid and rhabdoid dedifferentiation, which can occur across all histological subtypes, confers a particularly aggressive phenotype associated with higher tumor grades and poorer outcomes [2]. Tumor grading is performed using the World Health Organization/International Society of Urological Pathology (WHO/ISUP) nucleolar grading system (grades 1–4), which has largely replaced the Fuhrman grading system and is an independent prognostic factor for cancer-specific survival [2].

The propensity of RCC, and particularly ccRCC, for direct venous extension—rather than lymphatic spread—is a hallmark feature of its biology, with tumor invasion along the renal venous tributaries forming the venous tumor thrombus (VTT) that is the central focus of this review. The presence of venous tumor thrombus (VTT) in RCC fundamentally alters surgical complexity and patient prognosis, transforming a routine nephrectomy into one of urology’s most technically demanding procedures. Between 4–10% of RCC patients present as locally advanced disease, with tumor invasion into the venous system, which can further extend from the main renal vein(s) into the inferior vena cava (IVC), even reaching the right atrium in <1% of cases [3].

The presence of an IVC VTT portends a worse prognosis and significant surgical challenges. Indeed, about one-third of patients with an IVC thrombus have synchronous distant metastases at diagnosis [4]. Importantly, the cephalad extent of the thrombus correlates with surgical complexity and dictates the extent of interdisciplinary surgical planning required [4]. Untreated RCC with a VTT carries a dismal outlook—one study noted a median survival of only around 5 months without intervention [3]. Yet even these aggressive RCC phenotypes paradoxically maintain potential for curative treatment through complete radical surgical excision, with 5-year cancer-specific survival rates reaching 49% for limited renal vein involvement, and up to 26–40% for IVC extension in non-metastatic disease [4].

Open surgery remains the gold-standard approach for RCC with VTT, especially for thrombi extending into the infradiaphragmatic IVC [5]. In infrahepatic cases, the VTT is limited to the subhepatic IVC, which can most often be managed without cardiopulmonary bypass (CPB), whereas supradiaphragmatic extension typically necessitates cardiothoracic support. Minimally invasive laparoscopic or robotic techniques have been explored in select cases, but these are generally limited to lower-level thrombi and specialized centers, and long-term oncologic data are limited [6,7].

The evolution of surgical techniques for RCC with VTT reflects broader advances in vascular and transplantation surgery. Historical series from the 1970s reported perioperative mortality rates exceeding 13%, primarily due to massive hemorrhage and pulmonary embolism (PE) [8]. Contemporary approaches have dramatically improved these outcomes. The incorporation of liver transplantation techniques—notably “piggyback” liver mobilization, strategic use of vascular exclusion, and improved understanding of collateral circulation patterns [9]—has reduced perioperative mortality to <2% for infradiaphragmatic thrombi at high-volume centers [4,10]. It should be noted, however, that this benchmark derives predominantly from retrospective single-institution and multicenter case series rather than prospective registries, introducing potential reporting and selection bias inherent to high-volume center publications. Nevertheless, surgical resection of the kidney and VTT remains a complex endeavor with substantial morbidity; complication rates remain high, and mortality for thrombi extending above the major hepatic veins (MHVs) can reach up to 10% even in contemporary multicenter series [10], though this figure predominantly reflects the inclusion of supradiaphragmatic cases requiring CPB. These cases demand meticulous preoperative planning and multidisciplinary coordination at experienced centers (see Section 4).

The aim of this narrative review is to provide surgical urologists with a comprehensive, technically oriented, and evidence-based guide for the open surgical management of RCC with VTT confined to the infradiaphragmatic IVC (Mayo Levels 0–III). Specifically, we: (1) outline the preoperative evaluation pathway, including multimodal imaging for thrombus classification, assessment of IVC wall invasion, and patient optimization; (2) detail level-specific open surgical techniques for radical nephrectomy with en bloc IVC thrombectomy—encompassing operative planning, vascular control strategies, liver mobilization, cavotomy, thrombus extraction, and cavorrhaphy—supplemented by original intraoperative photographs and illustrative schematics; (3) review the evolving role of neoadjuvant and adjuvant systemic therapies in the perioperative setting; and (4) contrast open surgery with emerging robotic-assisted approaches. Level IV (supradiaphragmatic) thrombi requiring CPB are beyond the scope of this review.

## 2. Literature Search Strategy

This narrative review was conducted through a comprehensive search of the PubMed/MEDLINE, Scopus, and Web of Science databases. The following search terms were used in various combinations: “renal cell carcinoma,” “venous tumor thrombus,” “inferior vena cava,” “thrombectomy,” “radical nephrectomy,” “liver mobilization,” “piggyback,” “neoadjuvant,” “adjuvant,” “immunotherapy,” and “robotic.” The search encompassed articles published from January 1970 through March 2025, with no language restrictions. Reference lists of identified articles, current clinical practice guidelines (European Association of Urology [EAU] 2025 and National Comprehensive Cancer Network [NCCN] v2.2024), and established surgical atlases were also reviewed to identify additional relevant publications. The initial database search yielded circa 1500 records after deduplication. Following title/abstract screening, 200 full-text articles were assessed for eligibility, of which 101 met the inclusion criteria and informed the present review.

Titles and abstracts from the initial search results were screened by two authors/urologists (D.N. and S.L.) for relevance to the open surgical management of RCC with infradiaphragmatic VTT (Mayo Levels 0–III, as defined below). Full-text articles were then reviewed to confirm eligibility for inclusion. Studies were included if they reported on the preoperative evaluation, surgical technique, perioperative outcomes, or systemic therapy relevant to RCC with infradiaphragmatic IVC thrombus. Studies were excluded if they: (i) focused exclusively on supradiaphragmatic (Level IV) or intra-atrial thrombi managed with CPB; (ii) reported on non-RCC malignancies with IVC involvement; or (iii) were conference abstracts without full-text data available. Within the included literature, an evidence hierarchy was used to prioritize data extraction: prospective studies, randomized controlled trials, systematic reviews and meta-analyses, and large retrospective cohorts from high-volume centers were given precedence over smaller single-institution series. Case reports were included only when they illustrated novel techniques or outcomes not captured in larger series. As this is a narrative review, formal systematic review methodology (e.g., PRISMA) was not applied, and no quantitative data synthesis was independently performed.

## 3. Venous Tumor Thrombus Assessment and Characterization

### 3.1. Clinical Classifications

**Figure 1:**
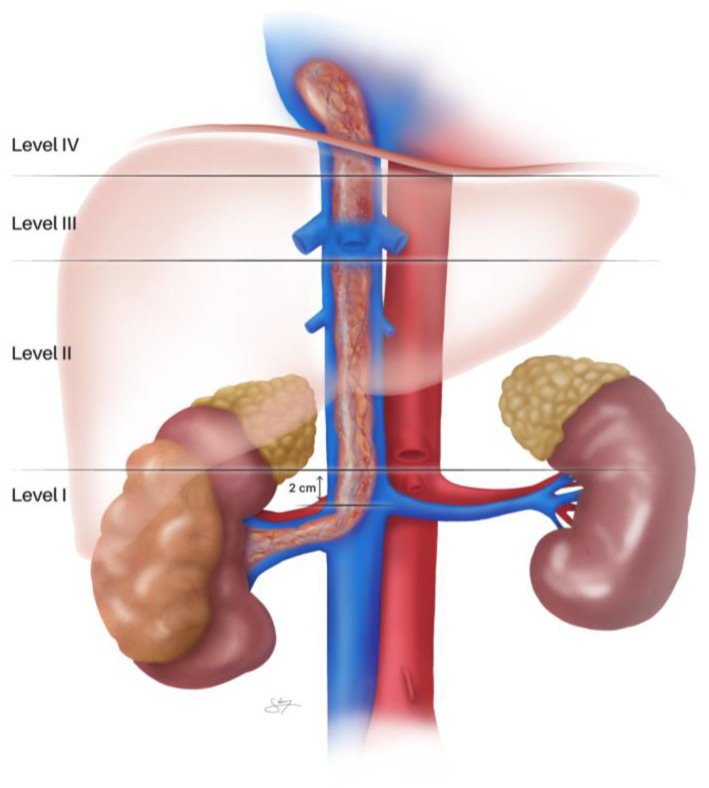
Schematic representation of the Mayo clinic classification of RCC Venous Involvement: Level 0—tumor thrombus confined to renal vein; Level I—thrombus extends into IVC ≤ 2 cm from the renal vein ostium; Level II—thrombus extends into infrahepatic IVC (below hepatic veins); Level III—thrombus extends into retrohepatic IVC up to but not above diaphragm (infradiaphragmatic); Level IV—thrombus extends above diaphragm or into the right atrium. N.B.: illustrated by our graphic design expert Silvia Claudia Dobre.

The Mayo Clinic classification system, originally described as a 4-level scheme by Neves and Zincke in 1987 [11] and subsequently expanded to five levels (0–IV) by Blute et al. in 2004 [4], is the most widely used system to categorize RCC venous thrombi by level, based on how far the thrombus extends from the renal vein upward [4,11]. This classification informs both prognosis and the anticipated surgical complexity [4,12], thereby providing the foundation for surgical planning and prognostication (Figure 1):

- Level 0: Thrombus confined to the renal vein (segmental or main renal vein) [4], representing the simplest scenario with surgical techniques similar to standard radical nephrectomy.
- Level I: Thrombus extends into the IVC but remains infradiaphragmatic and within 2 cm of the renal vein ostium [11], typically manageable through infrahepatic IVC control without liver mobilization.
- Level II: Thrombus extends farther into the infradiaphragmatic IVC, >2 cm above the renal vein, but still below the hepatic vein confluence [11], requiring more extensive IVC dissection and potential liver mobilization.
- Level III: Thrombus extends into the IVC above the hepatic vein confluence, but below the diaphragm (retrohepatic IVC) [11], necessitating complete liver mobilization and often hepatic vascular exclusion.
- Level IV: refers to thrombus extending above the diaphragm into the supradiaphragmatic IVC or right atrium of the heart—these RCC cases often require CPB support and are beyond the scope of this review.

Determining the thrombus level is crucial for operative planning [4,5]. For example, a level 0 or I thrombus may be handled with limited cavotomy and vascular control, whereas level II or III typically requires extensive IVC dissection and possibly liver mobilization. Modern imaging allows accurate classification in most cases, though final determination can sometimes be aided intraoperatively [13,14]. Other thrombus characteristics should also be assessed: whether there is wall invasion (direct tumor adherence to the IVC wall) or the presence of bland thrombus (blood clot) above or around the VTT. Tumor adherence to the endothelium and bland thrombi are associated with increased surgical complexity and risk of embolism [15,16]. In roughly 10% of cases, “descending” bland thrombus propagating caudally (e.g., into iliac veins) may coexist [15,17]. If extensive bland thrombus is present, prophylactic measures (such as placing a temporary IVC filter or careful intraoperative handling) may be considered to prevent pulmonary emboli, though venography is not routinely used due to its invasiveness [17,18].

The American Joint Committee on Cancer (AJCC) staging system [19,20] provides complementary classification, with T3a encompassing renal vein involvement, T3b indicating subdiaphragmatic IVC extension, and T3c denoting supradiaphragmatic spread or IVC wall invasion. This distinction proves critical as IVC wall invasion changes staging from T3b to T3c, conferring significantly worse prognosis with 5-year survival rates dropping from 37% to around 22–25% [9,21]. Recent evidence suggests subdividing level III into IIIa-d based on proximity to the diaphragm may better predict surgical complexity, though this modification awaits widespread validation [9,22].

### 3.2. Imaging Applications

Careful preoperative imaging is essential in the evaluation and surgical planning for RCC with VTT. Imaging serves multiple purposes: defining the extent and cranial level of the thrombus, assessing for vascular wall invasion, identifying “bland” thrombus (non-neoplastic, fibrin-rich clot often found adjacent to tumor extensions in the IVC), detecting venous collaterals, and screening for metastatic disease—all of which influence surgical approach and complexity.

Cross-sectional imaging of the abdomen and pelvis with contrast-enhanced multiphase CT or magnetic resonance imaging (MRI) constitutes the cornerstone of diagnostic workup. Multiphase contrast-enhanced CT with 2–3 mm slice thickness achieves 84% concordance with pathological findings for thrombus level classification [23,24]; the arterial phase at 25–30 s optimally demonstrates thrombus enhancement, while the nephrographic phase at 80–100 s delineates IVC involvement [24]. MRI provides superior accuracy for determining exact thrombus extent, particularly for Level III–IV cases, with 96–100% sensitivity [25,26], and is considered by many to be the gold standard for VTT assessment owing to its superior soft-tissue resolution for evaluating IVC wall invasion and diaphragmatic extension (i.e., MRI venography) [25,26].

**Figure 2:**
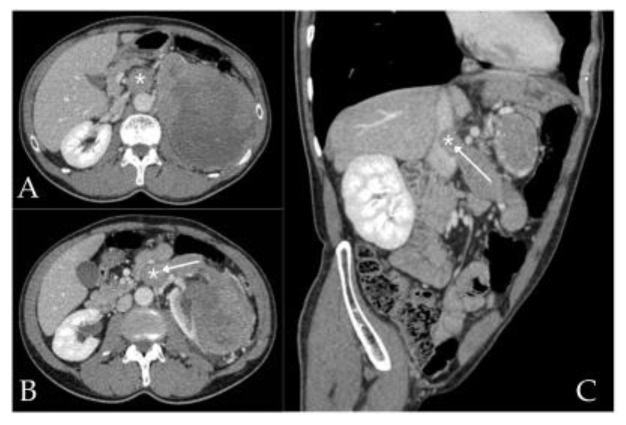
Contrast-enhanced computed tomography of locally advanced left-sided RCC (cT3bN0M0) with venous involvement (Level I)—multiple venous/nephrographic phase sections: (**A**) Axial (upper lumbar): large upper pole primary tumor (13/12 cm), with extensive intratumoral necrosis, and a significantly dilated left renal vein, occupied by the tumor thrombus (white asterisk); (**B**) Axial (lower lumbar): evident vascular invasion, with a displaced and markedly thicker left renal vein, molded around an extensive tumor thrombus (white asterisk), which is expanding towards the IVC from the left renal hilum (direction of white arrow); (**C**) Oblique (sagittal rotation in multi-planar reconstruction): expansion of tumor thrombus throughout the renal vein (direction of white arrow), reaching its peak cephalad extent (white asterisk) into the IVC lumen (≤2 cm above the ostium of the left renal vein).

Several imaging features predict IVC wall invasion: an IVC diameter exceeding 40 mm suggests extensive caval involvement [27], a renal vein ostium wall diameter exceeding 25 mm predicts ostial wall invasion requiring more aggressive resection [28], and complete IVC occlusion, irregular thrombus margins, and vessel wall thickening indicate probable wall invasion requiring potential vascular reconstruction [29,30,31]. A recent systematic review confirmed that both CT and MRI demonstrate high diagnostic accuracy, though a combined multi-technique workup is recommended for optimal assessment [32]. These modalities allow accurate visualization of the primary tumor (Figure 2A), renal vein and IVC (Figure 2B), and are highly effective in delineating the cranial extent of VTT (Figure 2C). However, most imaging accuracy studies are retrospective, single-institution analyses with inherent verification bias, and prospective, multi-institutional validation of imaging predictive models for IVC wall invasion remains lacking.

**Figure 3:**
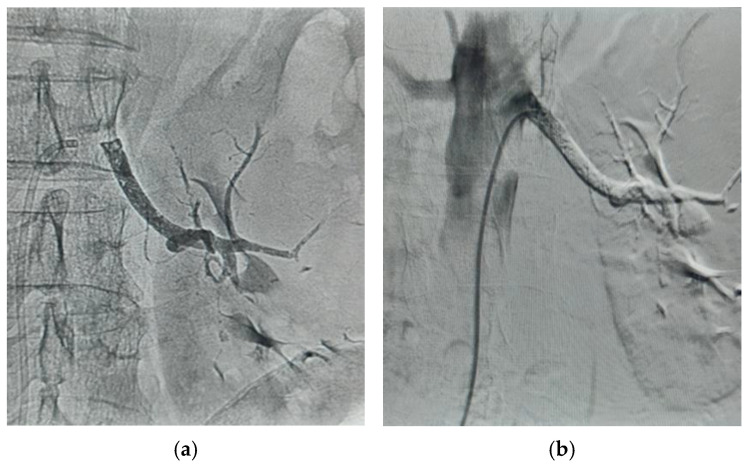
Preoperative interventional radiology angioembolization of left renal artery to facilitate surgical removal of a locally advanced left-sided RCC (cT3bN0M0) with venous involvement (Level I): (**a**) Left renal artery angiography before embolization; (**b**) Angiographic confirmation of successful left renal artery obstruction at aortic origin after embolization.

CT angiography provides additional vascular mapping, including renal arterial anatomy and IVC caliber, and is useful for planning vascular control. Also, preoperative interventional angiography (Figure 3a) and angioembolization of the renal artery (Figure 3b) may prove quite useful in achieving early intraoperative vascular control, especially when faced with a large tumor mass on the left side.

The EAU 2025 guidelines designate MRI as the imaging method of choice for thrombus extent, IVC occlusion, and wall invasion assessment [33], while the NCCN v2.2024 guidelines recommend MRI when thrombus extent is poorly defined on CT [34]. Imaging should ideally be repeated within 1–2 weeks of surgery given the potential for rapid VTT progression [33]. In practice, both MRI and modern multidetector CT are complementary and often used together in complex cases.

Although not part of the standard imaging workup, emerging data suggest a role for 18F-FDG PET/CT in specific clinical scenarios. PET/CT may help differentiate VTT from bland thrombus based on metabolic activity (VTT SUVmax significantly higher than bland thrombus; *p* < 0.001), though it remains inferior to contrast-enhanced MRI for thrombus level classification [35]. Furthermore, PET/CT metabolic parameters have shown promise in predicting IVC wall invasion preoperatively [36]. PSMA PET/CT has demonstrated approximately 85.7% avidity in clear cell RCC tumor thrombi, occasionally detecting thrombus extensions missed by conventional imaging [37]. However, the EAU 2025 guidelines continue to recommend against routine PET/CT for RCC staging [33], and its use in VTT assessment remains investigational.

Contrast-enhanced ultrasound (CEUS) has emerged as a valuable complementary imaging tool in the assessment of RCC with VTT. CEUS achieves high diagnostic accuracy for detecting bland thrombus within IVC VTT (sensitivity 87.5%, specificity 100%, accuracy 96.7%), information that directly impacts surgical strategy [38]. Intraoperative CEUS during IVC thrombectomy can differentiate bland from VTT in real time and detect IVC wall invasion, providing a useful adjunct to transesophageal echocardiography (TEE) [39]. Accordingly, the EAU 2025 guidelines now provide a strong recommendation (Level of Evidence 1b) for CEUS as a valuable alternative for further characterization of renal masses and thrombus when CT findings are indeterminate [33].

Three-dimensional (3D) reconstruction and virtual surgical planning represent emerging tools that may improve preoperative decision-making. A multicenter randomized controlled trial demonstrated that 3D-printed models for IVC thrombectomy planning led to significantly more patients requiring no ICU stay (OR 3.32), suggesting improved surgical preparedness [40]. First-in-human application of 3D augmented reality guidance during robotic IVC thrombectomy has also been reported, with precise intraoperative estimation of thrombus location in all cases [41].

Beyond VTT mapping, preoperative imaging must evaluate for metastatic disease, as the presence of distant spread (especially to lungs, brain, or bones) significantly influences treatment strategy. Chest CT is routinely performed to detect pulmonary metastases or tumor emboli, which may be present in up to 5% of RCCs with VTT [16,28]. Importantly, a PE at diagnosis is not considered a contraindication to surgery—studies suggest that it does not significantly worsen 90-day postoperative mortality or cancer-specific survival [42]. Brain imaging (CT or MRI) is indicated in RCCs with neurological symptoms to assess for intracranial metastasis, which may alter surgical timing/feasibility.

In patients with lower extremity edema, Doppler ultrasound is used to assess for distal venous thrombosis or IVC occlusion. Cystoscopic evaluation is occasionally warranted in cases of gross hematuria, to exclude concurrent urothelial carcinoma even when RCC is the dominant lesion.

Friable thrombi (with loose consistency) have been associated with higher risk of dissemination and worse prognosis [28,43], and if identified on imaging, underscore the need for careful intraoperative technique (to avoid fragmentation). If “bland” thrombus is suspected on imaging—typically identified as non-enhancing clot distinct from VTT—some clinicians advocate for preoperative anticoagulation or, in select cases, temporary IVC filter placement to reduce the risk of embolic events. However, filters are no longer routinely recommended due to potential complications during thrombectomy, such as entrapment or adherence to VTT.

Intraoperative imaging plays a critical adjunct role, especially for high-level thrombi. TEE, commonly employed before and during surgery, provides dynamic real-time monitoring of the thrombus tip and is particularly helpful in level III cases. TEE confirms that the thrombus remains infradiaphragmatic and detects any migration toward the right atrium. It also assists in identifying residual thrombus fragments or bland thrombus that might require additional control or influence the order of vascular clamping. In addition to the preoperative modalities detailed above, intraoperative CEUS can complement TEE by differentiating bland from tumor thrombus and detecting wall invasion in real time [39]. Together, the combination of preoperative cross-sectional imaging and selective intraoperative TEE and CEUS ensures comprehensive evaluation of the VTT before and during surgery.

## 4. Patient Evaluation and Perioperative Optimizations

The rationale for aggressive surgery is well established—patients can be cured if all tumor burden is removed. The ability of RCC to invade the IVC might suggest aggressive biology, yet many such tumors are still regionally confined (no metastases) and thus amenable to cure by resection [19,28]. Indeed, the juxtaposition of an intravascular VTT with an absence of distant spread in some patients represents a curious facet of RCC biology [17,31]. These observations justify undertaking major surgery despite the risks. The alternatives, observation or systemic therapy alone, yield poor outcomes in localized disease [44]. Therefore, in all patients with acceptable performance status and resectable disease, surgery should be strongly pursued.

Multidisciplinary planning is essential for these complex cases and is best conducted at high-volume centers, with a coordinated team involving urologic, vascular, and cardiothoracic surgeons on standby for higher-level thrombi. Hospital and surgeon volume have a profound impact on perioperative outcomes: a national database analysis demonstrated that high-volume institutions achieve a significant reduction in all-cause mortality (HR 0.76, *p* = 0.001) compared with lower-volume centers [45], and population-based data have shown that the majority of perioperative deaths occur within a surgeon’s first few thrombectomy cases [46]. These data strongly support centralization of IVC thrombectomy care to experienced multidisciplinary teams [45,46].

For Mayo Level III (retrohepatic) thrombi, planning for a complete intraoperative liver mobilization (a “hepatic transplant–type” full mobilization of the liver with division of the short hepatic veins [SHVs] to expose the retrohepatic IVC) and anticipating the possible need for CPB support (increasing exponentially if the thrombus extends aggressively upward, i.e., supradiaphragmatically towards the right atrium of heart), are obviously of paramount importance [12,28]. This being said, most infrahepatic IVC thrombi (Level I–III) can usually be managed without CPB, using strategic vascular control techniques [12,27]. However, patients must undergo thorough preoperative medical optimization and risk stratification. Close coordination with anesthesiology (for advanced hemodynamic monitoring and potential intraoperative TEE), and possibly perfusion teams, is emphasized to maximize safety.

### 4.1. General Considerations

Anesthetic management for IVC thrombectomy demands specialized expertise. Central venous access (typically via the internal jugular vein, avoiding femoral access due to potential IVC obstruction), arterial line monitoring, and large-bore peripheral intravenous access are mandatory. TEE is placed after induction and serves dual roles: continuous thrombus surveillance and real-time cardiac function assessment during cross-clamping. The anesthetic team must be prepared for rapid hemodynamic shifts—particularly during the test clamp phase and during thrombus extraction—requiring vasoactive infusions (norepinephrine, vasopressin) to be prepared and immediately available. Communication protocols between the surgical and anesthetic teams regarding the timing of clamping, unclamping, and any thrombus migration detected on TEE should be established before incision. For Level III cases, the availability of rapid transfusion devices (e.g., Level 1 or Belmont rapid infusers) is essential given the potential for acute, high-volume blood loss.

Comprehensive preoperative evaluation encompasses functional assessment and risk stratification. Performance status remains the strongest predictor of perioperative outcomes, with Eastern Cooperative Oncology Group (ECOG) scores of 0–1 identifying optimal surgical candidates. Beyond ECOG, frailty assessment is increasingly recognized as a complementary tool for patient selection. In a large national database study of patients undergoing radical nephrectomy, frail patients (modified Frailty Index ≥2) had significantly higher major complication rates (16.4% vs. 10.3%) and doubled mortality risk compared with non-frail patients [47]. A validated 15-point modified frailty index combined with ASA class has shown the best predictive accuracy for mortality and high-grade complications across major urologic oncology procedures [48]; however, no studies have specifically validated frailty indices in the IVC thrombectomy population, representing a knowledge gap deserving further investigation. All available frailty data are extrapolated from broader radical nephrectomy cohorts (Level of Evidence [LoE] 3b), and their direct applicability to the higher-acuity IVC thrombectomy population, which carries substantially greater hemorrhagic risk and hemodynamic stress, remains unvalidated.

Cardiac function assessment is especially critical in Level III cases that may require vascular bypass or extended hepatic vascular exclusion. Cardiopulmonary exercise testing (CPET), with established risk thresholds of anaerobic threshold < 11 mL/kg/min and peak VO2 < 15 mL/kg/min, may aid in risk stratification and ICU triage for these high-morbidity procedures, although no CPET data specific to IVC thrombectomy have been published [49]. Prehabilitation exercise programs have shown improvements in cardiorespiratory fitness before urologic cancer surgery, though impact on surgical outcomes has not yet been demonstrated [50].

Nutritional status warrants formal preoperative assessment. Preoperative albumin is among the most predictive markers for mortality and transfusion requirements in radical nephrectomy patients, and hypoalbuminemia (<3.5 g/dL) has been associated with early post-nephrectomy mortality [51]. The Prognostic Nutritional Index (PNI) has been validated in meta-analysis as an independent prognostic biomarker for survival after nephrectomy [52]. These data support incorporating nutritional screening and optimization into the preoperative pathway for IVC thrombectomy, particularly given the high hemorrhagic risk and prolonged recovery associated with these procedures.

Given the high hemorrhagic risk in these advanced RCC cases, ensuring ample blood products is critical and requires careful coordination with the institutional blood bank. Historical series report a mean estimated blood loss of around 1.8 L, with 62% of suprahepatic cases losing >2 L [53]. However, these figures vary substantially across series depending on thrombus level, surgical technique, and institutional experience.

Modern protocols recommend cross-matching 6–8 units of packed red blood cells, 4–6 units of fresh frozen plasma, and 2 units of platelets. Cell saver technology, despite theoretical concerns about tumor cell dissemination, has gained acceptance for reducing allogeneic transfusion requirements. A study specifically evaluating intraoperative cell salvage (IOCS) in patients with Level III–IV IVC thrombus undergoing radical nephrectomy with CPB found no increased local recurrence or distant metastases in patients receiving IOCS-salvaged blood; in fact, non-use of the cell saver was paradoxically an independent predictor of Clavien ≥ 3a complications (OR 18.71) [54]. These findings are corroborated by a meta-analysis of over 6300 subjects demonstrating that IOCS with leucocyte depletion filters actually reduces cancer recurrence risk (OR 0.71, 95% CI 0.58–0.86) compared with allogeneic transfusion alone [55]. Some centers also employ acute normovolemic hemodilution, removing 1–2 units of whole blood after anesthetic induction for reinfusion following tumor excision.

Conversely, anticoagulation management remains controversial and practice protocols continue to evolve. RCC patients with VTT carry a substantially elevated venous thromboembolism (VTE) risk: a large cohort study reported a 6.6-fold higher VTE risk compared with other RCC patients (22.4% two-year cumulative VTE incidence vs. 5.8% overall), with VTE occurrence significantly associated with worse survival [56].

Prophylactic anticoagulation is typically resumed approximately 12–24 h postoperatively, once hemostasis remains stable. Patients undergoing IVC ligation or extensive reconstruction often receive therapeutic anticoagulation with warfarin (targeting an INR of 2.0–3.0), though some centers report comparable outcomes without routine anticoagulation [27,57]. International guidelines recommend LMWH for initial VTE treatment in cancer patients, with LMWH preferred over direct oral anticoagulants for genitourinary malignancies due to increased bleeding risk [58,59]. Critically, no prospective data exist on the optimal type, dose, or duration of post-thrombectomy anticoagulation, representing an important knowledge gap [57]. Recent data suggest VTE rates of around 22% regardless of postoperative anticoagulation strategy [56], highlighting the need for individualized approaches based on the extent of IVC reconstruction and bleeding risk.

### 4.2. Neoadjuvant Systemic Therapy

The role of neoadjuvant systemic therapy is currently evolving, with preliminary trials having shown promising early results. Thereby, the NAXIVA Phase II trial of axitinib monotherapy reported down-staging of the thrombus level in 35% of patients, with a reduction in thrombus length in 75% overall and, despite non-responders, none of these patients demonstrated progression [60]. A 2025 translational analysis from NAXIVA further identified predictive biomarkers for axitinib response using machine learning (AUC 0.868 at baseline, 0.945 with week-3 features), including microvessel density and plasma cytokines, which may enable future patient selection for neoadjuvant TKI therapy [61].

Similarly, the NEOTAX trial demonstrated thrombus level reduction in 44% of patients receiving toripalimab plus axitinib, with a median thrombus length decrease of circa 2.3 cm [62]. Notably, NEOTAX—the first Phase II trial of combined immune checkpoint inhibitor (ICI) plus TKI specifically for IVC VTT—also reported that surgical strategy was changed in 62% of patients due to thrombus downstaging, and a pathological complete response (pCR) rate of 11.1% was achieved in the primary tumor [62]. Corroborating results have been reported from smaller studies evaluating tislelizumab plus axitinib [63].

Ongoing clinical research is exploring combination regimens (e.g., lenvatinib+pembrolizumab–NCT05319015; or pembrolizumab+axitinib–NEOPAX/NCT05969496), while some isolated reports have even documented complete VTT regression with immunotherapy alone [64]. Other retrospective series of tyrosine kinase inhibitors (TKIs) use have shown variable responses—some thrombi regressed, others remained stable [65]. Even so, while a reduction in thrombus extent might simplify surgery (potentially converting a level III to level II, obviating the need for bypass [12,28]), it is important to weigh the risks.

A comprehensive systematic review and meta-analysis encompassing 29 studies and 204 patients established the overall pooled thrombus level reduction rate at 29.4% with predominantly TKI monotherapy, and found that neoadjuvant therapy was associated with shorter operative time and less blood loss [65]. Even so, this estimate is derived predominantly from retrospective case series and single-arm phase II trials with significant heterogeneity in drug regimens, treatment duration, and response assessment criteria (LoE 3–4). Importantly, no survival benefit was demonstrated (cancer-specific survival *p* = 0.206, overall survival *p* = 0.442), though this may reflect the limitations of TKI monotherapy rather than the broader concept of neoadjuvant treatment [65]. ICI + TKI combinations appear to yield superior downstaging rates (44–62% in controlled phase II trials, up to 100% in case reports), compared to TKI monotherapy (29–35%). These figures should be interpreted cautiously, as the upper range derives from isolated case reports (LoE 5), while even the phase II data reflect single-arm designs without randomized comparator arms, and sample sizes remain limited (*n* = 18–36 per study).

Furthermore, considerable heterogeneity exists across neoadjuvant studies regarding patient populations (inclusion criteria ranging from any VTT level to Level II+ only, with variable inclusion of metastatic patients), drug regimen, treatment duration (range: 2–12 cycles), response assessment criteria (RECIST vs. thrombus-specific measurements), and timing of surgical intervention, limiting direct cross-study comparison. No randomized controlled trial has yet compared neoadjuvant ICI + TKI with upfront surgery in non-metastatic RCC with VTT. Additionally, all completed phase II trials enrolled highly selected patients at tertiary centers, introducing potential referral and selection bias that limits generalizability to broader clinical practice. Hitherto however, a 2025 propensity-score analysis has actually confirmed that preop immunotherapy does not significantly increase postop morbidity [66].

Even though systemic neoadjuvant approaches may potentially convert higher-level thrombi to more manageable cephalad IVC extensions, reducing operative complexity and blood loss, current guidelines still deem neoadjuvant therapy purely experimental/investigational in non-metastatic RCC with VTT. Thereby, neoadjuvant systemic treatments may be considered for clinical trials or in very specific scenarios (e.g., to facilitate a less morbid surgery in a borderline patient), yet they do not constitute a mandatory part of the standard of care. Conversely, vascular endothelial growth factor (VEGF)-targeted agents (TKIs), may cause adverse effects (such as arterial hypertension, hepatotoxicity, mucositis, and/or wound-healing complications) that might delay or complicate surgery. Thus, there is also an ethical concern with postponing a potentially curative surgical intervention in an advanced oncological patient, based on the currently incompletely characterized chance that the thrombus might shrink. As a result, the consensus is that surgery should not be delayed for systemic therapy outside a trial, given the risk of thrombus progression or embolization during treatment [33,60].

Multidisciplinary tumor board discussion is recommended if neoadjuvant therapy is contemplated. At our institution, neoadjuvant systemic therapy is reserved for highly selected cases (e.g., extensive Level III with bulky thrombus or oligometastatic disease where downsizing could meaningfully alter the therapeutic approach), and only within the framework of clinical trials. In all other patients without contraindications, we pursue upfront surgery, consistent with current guideline recommendations that prompt VTT removal offers the best chance for long-term survival [3,19,33]. This institutional approach reflects our interpretation of the available evidence and should not be generalized without consideration of local expertise and resources.

Modern perioperative protocols have dramatically reduced morbidity and mortality for IVC thrombectomy. Immediate postoperative care is tailored to thrombus level, with Level I–II cases often managed on standard surgical wards, while Level III–IV patients require intensive care monitoring for a mean duration of circa 4.4 days [53]. Continuous cardiac monitoring, Swan-Ganz catheterization for complex cases, and hourly urine output assessment are employed to detect complications early. Contemporary series report perioperative mortality < 2% for infradiaphragmatic thrombi, compared to 8–10% for Level IV cases requiring CPB support [28,67]. A recent national database analysis (2005–2022) confirmed that 30-day mortality ranges from 1.5–10% (2.0–3.3% at high-volume centers), with 90-day mortality of 5.8–6.6%, major complications (Clavien ≥ III) in 10–29%, and overall complication rates of 36–78% depending on VTT level and institutional volume [68].

Postoperative critical care is stratified by thrombus level and intraoperative course. Level II–III patients require ICU admission with continuous hemodynamic monitoring, hourly urine output assessment, serial arterial blood gas analysis, and coagulation profiles. Hepatic function panels (transaminases, bilirubin, alkaline phosphatase) should be monitored every 12 h for the first 48–72 h following procedures involving hepatic vascular exclusion. Postoperative ventilatory management may require extended intubation in patients with significant intraoperative volume resuscitation (>6 L crystalloid equivalent), and early extubation protocols should be adapted to the individual hemodynamic trajectory. Thromboprophylaxis with LMWH is typically resumed 12–24 h postoperatively once surgical hemostasis is confirmed.

Complications stratify predictably with thrombus level and surgical complexity. Major hemorrhage occurs in around 15–25% of patients, with 3–5% requiring reintervention for hemostasis control [28,53]. Even so, these rates reflect heterogeneous surgical approaches and patient populations. PE occurs in approximately 2–5% of cases intraoperatively, despite meticulous technique, with fatal intraoperative events in <1%, under modern preventive strategies [8,43]. Acute kidney injury requiring temporary dialysis develops in around 5–10%, while permanent dialysis dependence is seen in <2% [28,53]. Mean hospital stay length ranges from about 6.5 days for infrahepatic cases, to up to 11 days for cases with suprahepatic thrombi, though enhanced recovery protocols are progressively shortening these durations [53,68].

## 5. Open Surgery Techniques for Radical Nephrectomy with en Bloc Thrombectomy (Levels 0–III)

Open surgical management of RCC with VTT requires strict adherence to sound vascular and oncological safety principles: adequate exposure, proximal and distal control of blood flow, en bloc tumor removal, and careful closure to prevent hemorrhage or embolism. The VTT level guides the complexity and extent of the operative approach, ranging from a simple extension of standard radical nephrectomy for Level 0 disease to a multistep operation involving complete liver mobilization and hepatic vascular exclusion for Level III thrombi [5,69]. The unifying goal across all levels is complete resection of the kidney and of the entire VTT as a single, intact specimen, while minimizing the risk of intraoperative tumor dissemination and catastrophic hemorrhage.

Below, we outline the standard operative steps and key technical considerations for each thrombus level. While detailed procedural descriptions are provided to serve as a practical technical reference, we emphasize evidence-based comparisons between alternative management strategies at each decision point—including the choice of incision, vascular control technique, cavotomy approach, and need for circulatory support—drawing on the available comparative data and institutional series, as highlighted below.

### 5.1. Patient Positioning and Operative Exposure

Open radical nephrectomy with thrombectomy is typically performed via a transperitoneal approach. The patient is positioned supine, with slight lumbar extension to optimize exposure; this position also offers advantages for anesthetic and surgical control, allowing better access to the patient’s head and chest when TEE or CPB are required [70]. A generous abdominal incision is essential to provide adequate access to both the renal hilum and the entire involved segment of the IVC. A bilateral subcostal (chevron) incision or midline xipho-pubic laparotomy are most commonly employed, as both provide wide retroperitoneal access without requiring thoracic extension [5,69,70]. The chevron incision is particularly suited for right-sided tumors requiring liver mobilization, while a midline incision offers versatility for left-sided tumors and can be extended cephalad via sternotomy if CPB becomes necessary. The triradiate chevron incision combines the advantages of both approaches without increasing incision-related complications [70]. Regardless of incision choice, thoracic extension is generally unnecessary for infradiaphragmatic thrombi (Levels 0–III), as even Level III disease can usually be managed via an abdominal-only approach [5,69].

After abdominal entry, exposure is secured using a self-retaining retractor system (e.g., Omni-Tract or Bookwalter for midline incisions; Rochard or Thompson liver retractors for chevron incisions) [70]. The ipsilateral colon is mobilized medially along the white line of Toldt—reflecting the right colon for right-sided tumors, or the left colon and splenic flexure for left-sided tumors—to expose the retroperitoneum. For right-sided tumors, the posterior peritoneum is incised lateral to the ascending colon; the right colon is reflected medially and the duodenum is “kocherized” (Kocher maneuver) to expose the anterior surface of the IVC and aorta (Cattell–Braasch maneuver) [53,69]. On the left side, mobilization of the descending colon is followed by en bloc mobilization of the spleen, pancreas, and stomach toward the midline to fully inspect the left anterior renal plane and expose the renal hilum and left renal vein as it crosses over the aorta [70]. It should be noted that left-sided tumors with IVC VTT are generally more technically challenging, as the IVC is most accessible from the right retroperitoneum while the tumor and renal hilum lie on the left; for this reason, bilateral colonic mobilization and wider exposure are often required, and the midline and chevron incisions provide the best access for these cases [5,69]. These visceral mobilization steps are essential to achieve adequate working space for vascular control of the great retroperitoneal vessels. At this stage, the kidney is only partially mobilized and remains attached by its vascular pedicle until vascular control of the IVC is fully secured, to minimize the risk of VTT embolization during manipulation [53].

### 5.2. Vascular Control

#### 5.2.1. Early Renal Artery Ligation

A critical early step is control of the ipsilateral renal artery before addressing the venous thrombus. Early ligation or clipping of the renal artery reduces arterial inflow, decompresses venous collaterals, potentially limits blood loss later in the case, and may permit retraction of the cephalad extent of the VTT [5,69,71]. For right-sided tumors, the renal artery is best approached in the interaortocaval space, which decreases early manipulation of the IVC and right renal vein [4]. For left-sided tumors, the artery is identified in the paraaortic region. The artery is secured with a 2-0 silk ligature or a large clip [69].

Access to the main renal artery can be achieved through either an anterior approach (requiring full mobilization of the peritoneal structures to enter the retroperitoneum) or a posterior approach (en bloc mobilization of the kidney with the peritoneal structures using Cattell–Braasch and Mattox maneuvers, creating a plane of cleavage anterior to the posterior abdominal wall) [70]. The posterior approach avoids potentially engorged venous collaterals on the anterior renal surface, providing quick and safe access to the main renal artery near its aortic takeoff, and may represent the best option in cases of marked venous collateral circulation [70].

For left-sided tumors, especially those with large masses, preoperative interventional angioembolization of the renal artery (Figure 3) may prove useful in achieving early intraoperative vascular control and reducing bleeding from collateral vessels. In approximately one-third of cases, tumor thrombi have an independent blood supply arising from the renal artery or the aorta, and angiographic infarction of this blood supply can help shrink a large thrombus, sometimes allowing bypass and/or hepatic mobilization to be avoided [69].

#### 5.2.2. IVC and Renal Vein Control

Attention then turns to achieving complete vascular control of the IVC and renal veins. The IVC is meticulously dissected circumferentially both below and above the thrombus level. All lumbar vein branches and any additional tributaries within the involved segment are identified, ligated, and divided to allow full mobilization of the cava. Every vestige of lymphatic tissue should be cleared from the anterior aspect of the infrahepatic IVC segment; the posterior surface of the IVC must be detached from the posterior abdominal wall by ligating and dividing all lumbar veins at this level, thus gaining complete circumferential control. This step should be performed with great care, given that lumbar venous vessels may be engorged in response to IVC occlusion, and uncontrolled bleeding at this level can be extremely dangerous [70].

**Figure 4:**
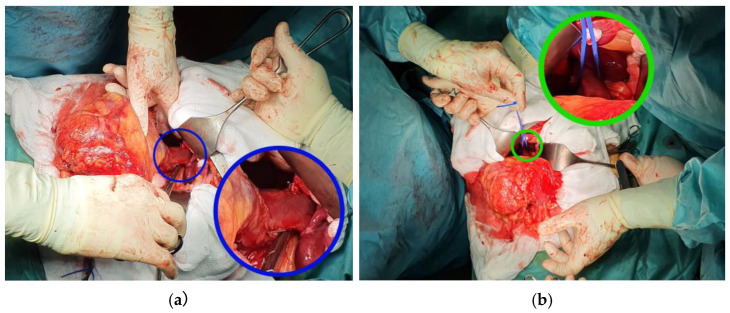
Complete circumferential IVC dissection cephalad of maximum tumor thrombus extent—live surgery (right-sided RCC, Level I): (**a**) Overholt clamp passed posterior to the IVC, demonstrating complete circumferential mobilization (green circle highlights the clamp tip emerging behind the IVC); (**b**) Tourniquet loop (blue vessel loop) passed behind the IVC for vascular control.

In addition, the ipsilateral gonadal vein (the right gonadal vein on the anterior IVC surface, or the gonadal branch of the left renal vein) and adrenal vein(s) within the dissection field must be identified and ligated. For left-sided tumors, the adrenal, lumbar, and gonadal branches of the left renal vein are frequently dilated and friable due to venous congestion and may occasionally harbor bland or tumor thrombus—frozen section should be obtained if suspicion arises [69]. The right adrenal vein, a short vessel draining directly into the IVC, requires particular attention during right-sided Level II–III dissections to avoid avulsion injury [5,69]. Complete circumferential IVC dissection before any clamping is essential to prevent catastrophic posterior wall injury from unsecured lumbar veins. The contralateral renal vein is also dissected free. Figure 4 demonstrates the technique of circumferential IVC dissection cephalad of the maximum VTT extent, showing the passage of an Overholt clamp behind the IVC (Figure 4a) and a tourniquet loop being passed behind the IVC to achieve control (Figure 4b).

Once circumferential control is achieved, vascular clamps or Rummel tourniquets are sequentially placed. Vessel loops can be passed through a short (7.5–15 cm) 18-Fr red rubber catheter and used as Rummel tourniquets; these are preferred over vascular clamps as they are more easily adjustable and less likely to pinch and fracture the VTT [69]. The sequential clamping order is: (1) infrarenal IVC, (2) contralateral renal vein, and (3) suprarenal IVC just cephalad to the tumor’s upper extent [5,53,69]. This establishes complete venous occlusion, isolating the segment containing the thrombus. It should be noted that no randomized trials have compared Rummel tourniquets with vascular clamps for IVC control during thrombectomy; the preference for tourniquets is based on expert consensus and retrospective institutional experience suggesting lower rates of VTT fragmentation [69]. Similarly, the optimal sequential clamping order has not been formally evaluated in comparative studies, though the infrarenal-first, suprarenal-last sequence is uniformly recommended across published surgical atlases and institutional series [5,53,69].

**Figure 5:**
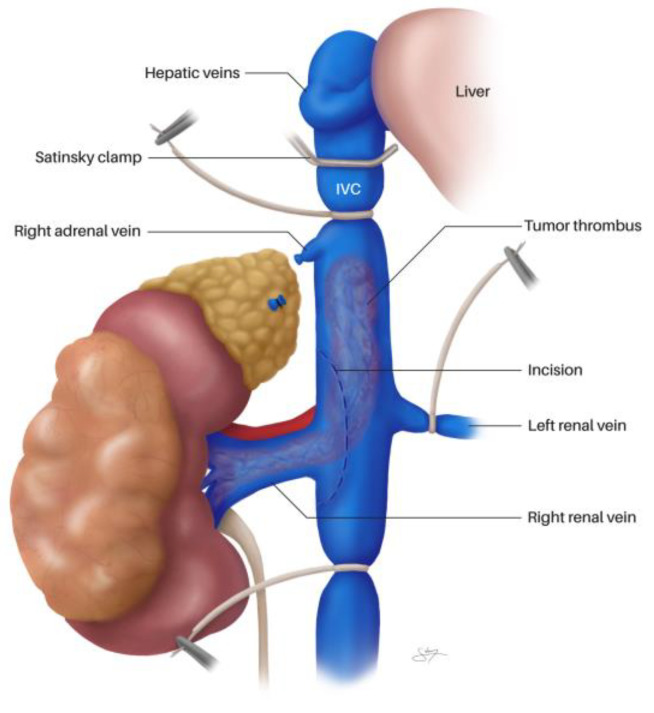
Illustration of complete vascular control in level II thrombus. Tourniquet on distal IVC, proximal IVC cephalad of maximum tumor extent and contralateral renal vein. Satinsky clamp placed above tourniquet for additional control, above maximum tumor extent. Lateral cavotomy line is represented. N.B.: illustrated by our graphic design expert Silvia Claudia Dobre.

Figure 5 illustrates the overall scheme of vascular control, with tourniquets on the distal IVC, proximal IVC cephalad of the maximum tumor extent, and the contralateral renal vein; a Satinsky clamp is placed above the tourniquet for additional control, and the lateral cavotomy line is represented.

Optionally, the contralateral renal artery can be clamped to prevent renal engorgement while venous outflow is temporarily occluded—this is more of an issue for left-sided tumors, since the right kidney does not have significant venous collateralization to shunt blood when the right renal vein is clamped. Prior to clamping, some surgeons administer 0.5 mg/kg of intravenous heparin to prevent clamp-related thrombotic complications [69].

#### 5.2.3. Test Clamp and Hemodynamic Assessment

Before proceeding to cavotomy, a “test clamp” of the IVC is recommended to ensure hemodynamic stability [5,69]. The IVC is occluded above the VTT and the patient’s hemodynamic response is observed over 2–5 min. Clamping the suprahepatic IVC produces significant hemodynamic changes: a 60% reduction in cardiac preload, an 80% increase in peripheral vascular resistance, a 50% increase in heart rate, a 40% drop in cardiac output, and a 10–20% drop in mean arterial blood pressure (MAP). Hemodynamic thresholds for intolerance include a cardiac output drop exceeding 50% or a MAP drop exceeding 30%, either of which should prompt consideration of veno-venous or CPB [69]. Patients with free-floating, partially occlusive thrombi generally do not tolerate suprahepatic clamping, whereas patients with completely occlusive thrombi typically have developed extensive collateral venous drainage networks and therefore tolerate clamping much better [69].

In most infradiaphragmatic cases, venous return is maintained via collateral pathways (lumbar–azygos system and portal circulation), allowing surgery to continue without bypass. However, if clamping a fully patent IVC with minimal collateral development precipitates severe hypotension, veno-venous bypass may be needed to augment venous return. Throughout these maneuvers, the anesthesia team should be vigilant for any VTT embolization. Intraoperative TEE (described in detail in Section 3.2) is routinely employed for real-time monitoring of the thrombus position, detection of migration, and cardiac function assessment during cross-clamping [5,53,69,70]. Intraoperative TEE is recommended for all Level II–IV thrombi [69].

### 5.3. Liver Mobilization for Level II–III Thrombi

For Level II thrombi (infrahepatic IVC, below the hepatic veins), vascular control as described above is usually sufficient. However, division of the caudate lobe veins (short hepatic veins [SHVs]) draining into the retrohepatic IVC can be helpful when the thrombus approaches the hepatic vein inflow, as this frees an additional 2–5 cm of cephalad IVC length and facilitates clamp placement just below the MHVs [5,53].

Level III thrombi (extending into the retrohepatic IVC above the hepatic veins but below the diaphragm) present greater technical challenges and typically demand more extensive liver mobilization using techniques borrowed from liver transplantation surgery [5,70,72]. The thrombus level reached inside the IVC determines the extent of liver dissection. Classical mobilization of the right hepatic lobe (Langenbuch maneuver), involving division of the right triangular and coronary ligaments, allows gradual rotation of the liver toward the midline and access to the right lateral surface of the IVC. However, this maneuver may be insufficient when circumferential IVC control is needed and CPB is not planned.

For more proximal thrombi, full liver mobilization using the “piggyback” technique—first described by Tzakis et al. in 1989 for liver transplantation—enables total vascular control on the abdominal segments of the IVC behind and above the liver [70]. With the piggyback technique, the liver is fully mobilized by dividing all of its attachments: the ligamentum teres hepatis (remnant of the obliterated left umbilical vein, located at the lower free border of the falciform ligament) is ligated and divided; the falciform ligament is divided with electrocautery up to the upper border of the liver, where it branches into the coronary ligament (on the right) and left triangular ligament (on the left) [69]. The superior layer of the coronary ligament is divided, continuing along the right border of the liver until the right triangular ligament is reached and divided. The inferior layer of the coronary ligament is then divided upward toward the IVC. The left triangular ligament is divided anteriorly and posteriorly toward the IVC to complete hepatic mobilization [69]. The right lobe of the liver can now be safely and gently rotated toward the midline so that the IVC can be evaluated on the posterior surface of the liver.

**Figure 6:**
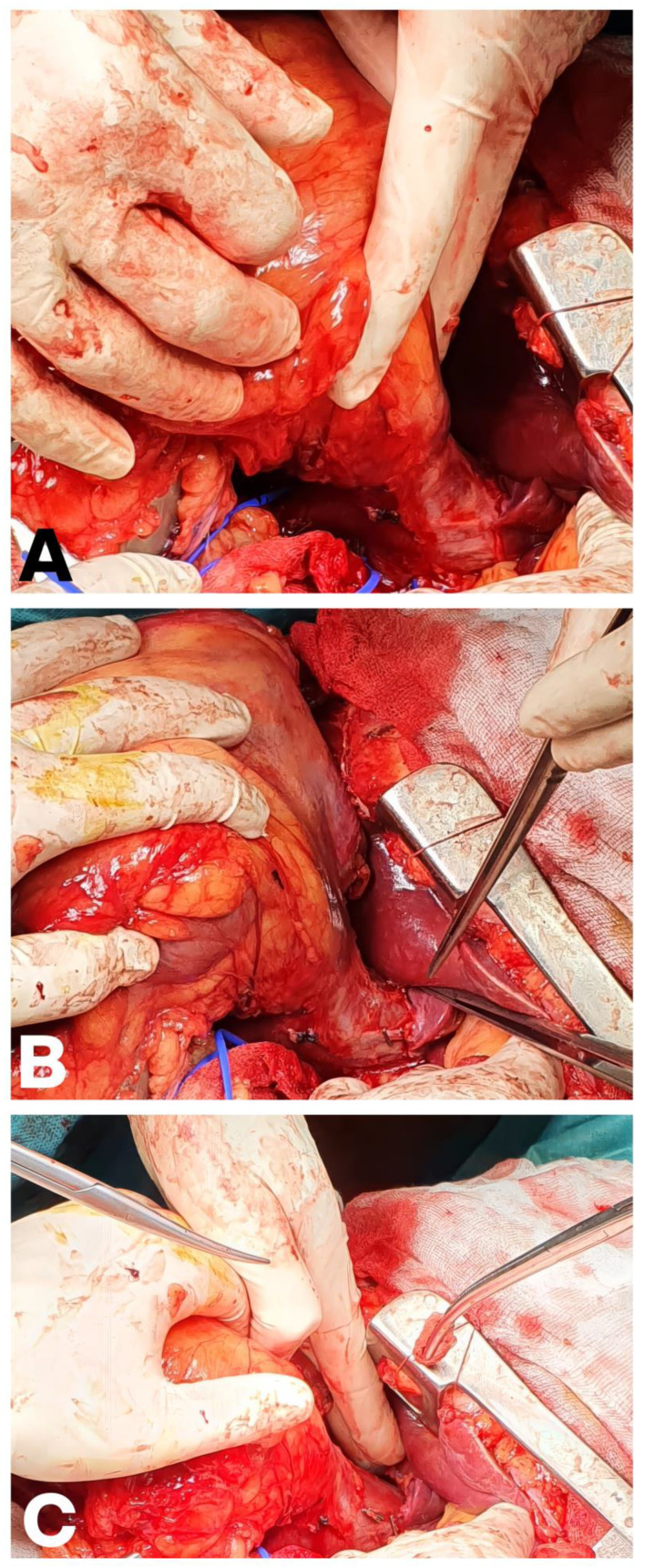
Intraoperative images of liver mobilization in level II thrombus: (**A**) Dissected short hepatic veins (SHVs); (**B**) Clipped SHVs; (**C**) Incised SHVs and continued cephalad dissection.

A plane between the posterior surface of the liver and the anterior surface of the IVC must then be developed [69,72]. This plane contains venous branches (SHVs) from the liver, which are divided into the upper and lower groups. The SHVs are individually identified, dissected, clipped, and divided, continuing the cephalad dissection until the liver is fixed to the IVC only by the MHVs [70]. This critical sequence of steps is demonstrated in Figure 6, which shows the dissected SHVs (Figure 6A), the clipped SHVs (Figure 6B), and the incised SHVs with continued cephalad dissection (Figure 6C). Once complete, the liver can be “softly rolled” to the left side of the peritoneal cavity while preserving hepatic venous drainage through the MHVs, providing unprecedented access to the retrohepatic and suprahepatic IVC and facilitating circumferential clamping cephalad to the most proximal extent of the VTT.

The aid of a hepatobiliary or transplant surgeon may be valuable for this portion of the procedure [5,69]. With the liver mobilized, the lesser omentum is opened to permit isolation and control of the porta hepatis (portal triad: portal vein, common hepatic artery, and common bile duct) with a Rummel tourniquet, enabling a Pringle maneuver if needed [69]. The Pringle maneuver—temporary occlusion of the portal venous and arterial inflow of the liver—prevents hepatic venous congestion and the attendant risk of hepatic capsule fracture and hemorrhage while the hepatic veins are clamped [69,70]. Clamping the IVC above and below the hepatic veins while performing a Pringle maneuver constitutes total hepatic vascular exclusion (HVE) [69,70]. Under normothermic conditions, the porta hepatis can be clamped for up to 60 min, although a clamping time of 20 min or less is preferred because ischemic hepatic injury and portal vein thrombosis can result [69]. Sequential 15-min periods separated by 5-min reperfusion intervals can extend this window. A potential complication of the Pringle maneuver is splenic engorgement and rupture due to backup of venous drainage from the splenic vein [69].

If the VTT can be milked down below the hepatic venous confluence, a tourniquet may instead be applied to the IVC below the hepatic venous outflow, thereby avoiding the need for the Pringle maneuver and hepatic congestion [5,69]. Ideally, the IVC should be clamped below the MHVs because the venous return from the liver is considerable [69]. However, for thrombi that extend into or above the hepatic veins and cannot be milked down, a suprahepatic clamp with HVE is necessary.

An alternative technique for proximal IVC control, particularly when circumferential dissection of the retrohepatic IVC is difficult, involves insertion of an intracaval Fogarty balloon catheter (9-Fr) through a small cavotomy, advanced past the thrombus under real-time TEE guidance (see Section 3.2) and inflated to achieve temporary intrahepatic or suprahepatic IVC occlusion [69]. This approach avoids the need for complete retrohepatic dissection in select cases and can serve as a useful adjunct to the standard clamping techniques described above.

In some instances, especially for high-reaching Level III thrombi, transdiaphragmatic control of the IVC is employed: an incision in the central tendon of the diaphragm (and pericardium if necessary) allows passage of a tourniquet or Satinsky clamp around the intrapericardial IVC above the thrombus [53,73]. The absence of tributaries makes circular mobilization of the intrapericardial part of the IVC significantly easier compared with subdiaphragmatic circular mobilization [53]. This technique achieves suprahepatic control without requiring a formal thoracotomy or CPB and can often be performed by the abdominal surgical team alone.

Hemodynamic instability may result from the combination of IVC cross-clamping and the Pringle maneuver; accordingly, the test clamp protocol described in Section 5.2.3 should be performed before proceeding with cavotomy. In the event of significant hypotension, vascular bypass via either CPB with hypothermic cardiac arrest or veno-venous bypass is indicated. Nevertheless, most infradiaphragmatic Level III thrombi can be managed without full CPB at experienced centers, with veno-venous bypass serving as a useful intermediate option when hemodynamic stability cannot be maintained with clamping alone. The team must, however, be prepared for rapid escalation to bypass if needed. Close coordination with cardiac and/or vascular surgeons is ideal for all Level III cases.

### 5.4. Cavotomy and Thrombectomy

After achieving vascular occlusion, the next phase is removing the VTT in continuity with the kidney. The cardinal goal is en bloc resection: the kidney, renal vein, and VTT are extracted as one intact specimen to avoid fragmentation and minimize the risk of dissemination.

#### 5.4.1. Level 0 and Level I Thrombi

For low-level thrombi (Level 0, confined to the renal vein, or Level I, extending into the IVC ≤ 2 cm above the renal vein ostium), a formal longitudinal cavotomy is generally unnecessary. Level I IVC thrombi are typically partially occlusive and nonadherent, and do not require extensive IVC dissection or any form of bypass [69]. They can routinely be treated in a similar fashion to Level 0 thrombi: reduced into the renal vein, encompassed with an appropriately shaped vascular clamp, and removed in continuity with the kidney and renal vein [5,69,70].

Following early arterial ligation, the kidney is gently mobilized outside the renal fascia, the ureter is divided, and the IVC is dissected above and below the renal vein [69]. The surgeon places a hand on the IVC, gently pinches it closed starting as cranially as possible, and milks the IVC toward the ostium of the renal vein. A C-shaped Satinsky vascular clamp is placed around the ostium of the renal vein, approaching from lateral to medial and partially occluding the IVC. This maneuver ensures the thrombus is located entirely within the jaws of the clamp before closing it, thereby preserving blood flow through the IVC and preventing embolization of dislodged thrombus fragments to the pulmonary circulation [69,70]. Full IVC cross-clamping is generally unnecessary for Level 0 if the thrombus is small; however, if the thrombus is mobile or extends just beyond the ostium, full IVC cross-clamping below and above the thrombus is recommended to avoid any risk of dissemination during manipulation.

**Figure 7:**
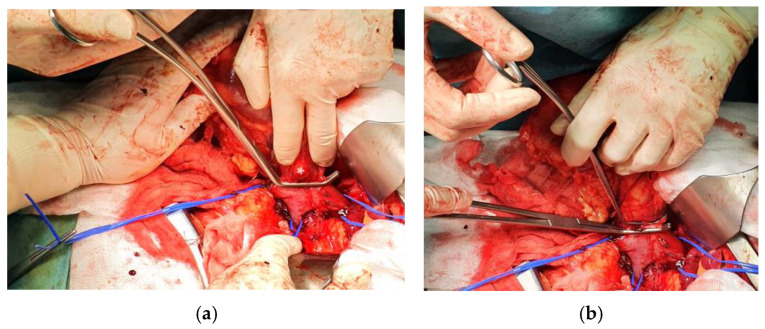
Lateral clamping of the IVC at the level of the right renal vein, beyond the tumor thrombus’ peak cephalad extent—live surgery: (**a**) First Satinsky clamp applied to ‘milk’ the thrombus toward the renal vein ostium (white asterisk [*] marks the renal vein ostium); (**b**) Second Satinsky clamp secured caudally toward the excision specimen, isolating the thrombus-bearing segment between the two clamps.

In some Level I cases, the thrombus is too bulky or friable to be completely reduced into the renal vein by milking alone, yet does not extend far enough into the IVC to warrant the extensive mobilization required for a formal longitudinal cavotomy. In this scenario, the “milking” maneuver is combined with a limited lateral cavotomy. Once the thrombus has been milked as far as possible toward the renal vein ostium, a Satinsky clamp is placed on the IVC just cephalad to the renal vein insertion, using it to gently compress the IVC against the thrombus and displace it further toward the ostium. A second Satinsky or vascular clamp is then applied on the cava just caudal to the renal vein, effectively trapping the thrombus segment between the two clamps. This two-clamp technique prevents the thrombus from slipping during the venotomy [70]. Figure 7 demonstrates this lateral clamping maneuver intraoperatively in a Level I case: the first Satinsky clamp is used to “milk” the thrombus toward the renal vein (Figure 7a), and the second clamp is then secured toward the excision specimen to isolate the thrombus-bearing segment (Figure 7b).

**Figure 8:**
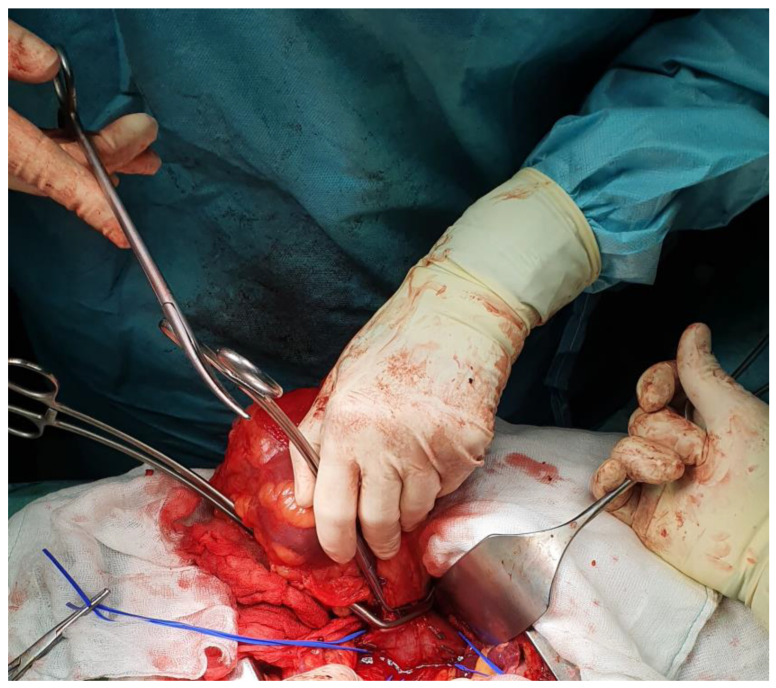
Intraoperative image of lateral cavotomy—the surgical scissor is positioned to incise the right anterolateral IVC wall between the secured proximal and distal Satinsky clamps. The blue vessel loop visible inferiorly marks the contralateral renal vein.

The right lateral aspect of the IVC is then incised longitudinally between these clamps to enter the lumen—a minimal lateral cavotomy that provides sufficient access for thrombus extraction without the wider incision required for higher-level thrombi. Figure 8 captures this moment: the surgical scissor is poised to create the lateral incision between the secured Satinsky clamps.

Whether the approach involves a simple ostial circumscription or a limited lateral cavotomy, the IVC is palpated for evidence of additional thrombus. Laparotomy sponges are placed around the renal vein to catch any malignant cells that may drip from the open renal vein. The renal vein ostium is then circumferentially incised using a scalpel, fine-tip Metzenbaum scissors, or Potts scissors, taking care not to cut away too much IVC wall or to incise into VTT [69]. The thrombus is extracted intact by gentle downward traction on the renal vein; a gauze is wrapped around the renal vein stump and thrombus and secured with a silk ligature to prevent tumor spillage [69]. This permits removal of the VTT en bloc with the nephrectomy specimen and attached renal vein.

Following thrombus extraction, the IVC is inspected for evidence of residual VTT, and its lumen is irrigated with heparinized saline solution (100 U/mL) for improved visualization [69]. The IVC defect is closed with a running 4-0 polypropylene suture on a vascular needle. Prior to tying the knot, the infrarenal IVC clamp is released and 5–10 mL of blood is allowed to seep from the cavotomy to flush out any residual thrombus fragments, air, and debris before the closure is completed [5,69].

#### 5.4.2. Level II and III Thrombi: Cavotomy Techniques

For higher-level thrombi (Levels II–III with more extensive IVC involvement), an IVC cavotomy is required. If the IVC clamping trial is tolerated and the thrombus can be removed in less than 30 min, it is safe to proceed with the intraabdominal procedure [69]. The clamps are applied in the established sequence: infrarenal IVC, contralateral renal vein, porta hepatis (if performing HVE), and suprahepatic IVC. For left-sided tumors, the right renal artery should be clamped prior to the right renal vein since the right kidney lacks significant collateral venous drainage [69].

There are two principal techniques for the cavotomy: a longitudinal midline (anterior) cavotomy versus a lateral cavotomy (incising the right anterolateral wall). A midline cavotomy provides a larger opening—useful for bulky or adherent thrombi—but involves a greater portion of the caval wall. A lateral cavotomy creates a smaller defect that can be easier to repair and, in many cases, offers sufficient access for thrombus extraction. An “L”-shaped cavotomy, performed longitudinally along the isolated IVC and extending over the renal vein ostium, is another well-described approach for Level II thrombi that combines the advantages of both orientations [4,5]. No comparative studies have formally evaluated the relative merits of midline versus lateral cavotomy; the choice remains guided by surgeon preference, thrombus extent/morphology, and the degree of anticipated wall invasion/adherence. The lateral approach is generally favored in published series for its smaller wall defect and simpler repair [5,69,70], while the midline approach is reserved for bulky or adherent thrombi requiring maximal exposure.

**Figure 9:**
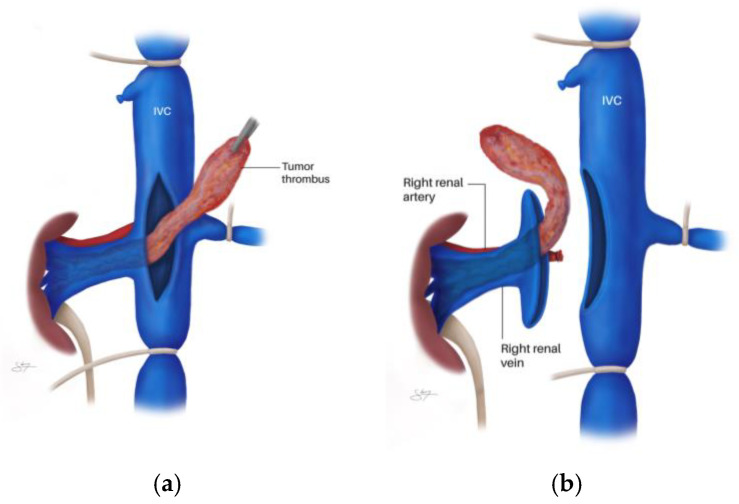
Illustration of cavotomy techniques: (**a**) Midline cavotomy with thrombus extraction for more extensive involvement; (**b**) Lateral cavotomy with efficient thrombus “milking”. N.B.: illustrated by our graphic design expert Silvia Claudia Dobre.

Figure 9 illustrates both principal cavotomy approaches side by side in a conceptual schematic: a midline cavotomy with thrombus extraction for more extensive involvement (Figure 9a), providing excellent visibility and room for extraction though creating a larger defect, and a lateral cavotomy with efficient thrombus “milking” toward the renal vein ostium (Figure 9b), which is preferred when the thrombus can be effectively displaced downward. In practice, the lateral approach is favored when anatomy permits, as it creates a smaller IVC wall defect, facilitates easier primary cavorrhaphy, and minimizes the risk of significant IVC narrowing.

The thrombus is grasped and carefully dissected free from the IVC wall with a combination of blunt and sharp maneuvers. Any fibrous adhesions of tumor to the endothelium are sharply divided. A Penfield dissector is used to help clear the IVC of adherent thrombus, and a 20-Fr Foley catheter can be utilized as an embolectomy catheter if thrombus is out of reach [69]. Once freed, the VTT is delivered en bloc with the kidney. In a right-sided tumor, this typically involves excising the right renal vein flush with the cava (or an “ostial cuff” of cava around the vein) so that the kidney, renal vein, and thrombus come out together as one unit. For a left-sided tumor, the left renal vein may be divided early (after clamping) to facilitate thrombus retrieval; the thrombus is then removed from the IVC and the specimen includes the left renal vein stump containing the tumor.

#### 5.4.3. Two-Step Cavotomy for Retrohepatic Thrombi

In cases where the VTT extends into the retrohepatic IVC and cannot be milked down below the level of the major hepatic vein ostia, a two-step cavotomy may represent the best option. This process requires temporary hepatic vascular exclusion (HVE), performed as described in Section 5.3. In the first step, the IVC wall is opened to a level below the MHVs, and the lumen is flushed with heparinized saline and completely cleared of thrombus fragments up to this level. The proximal clamp is then repositioned below the MHVs, and the IVC wall is closed with a running 4-0 polypropylene suture. The Pringle maneuver is then released (safe hepatic ischemia limits per Section 5.3), restoring natural hepatic venous bypass. In a second step, the cavotomy is continued downward to the renal veins, and every vestige of neoplastic tissue is withdrawn from the IVC lumen. Commonly, the caval wall containing the renal vein ostium with tumor involvement is also excised to ensure a safe resection margin [70].

Throughout the thrombectomy, meticulous technique is essential to avoid embolization: the thrombus is handled gently, and any bland clot extending below or above the tumor is removed. It is not uncommon to encounter a bland thrombus in the infrarenal IVC segment; if present, this should be carefully extracted. If a chronic infrarenal occlusion cannot be fully cleared, one option is to ligate the cava below the renal veins to prevent postoperative pulmonary emboli from residual clot. The Mayo Clinic classification of concomitant bland thrombus further refines the surgical approach: Group A (no bland thrombus); Group B (bland thrombus limited to pelvic veins—manageable with an intraoperative infrarenal IVC filter); Group C (diffuse infrarenal bland thrombus—best managed by permanent IVC interruption); and Group D (retrograde tumor thrombus growth admixed with bland thrombus—requiring segmental IVC resection below the contralateral renal vein ostium to maximize the chance of complete clearance) [17,69]. Maximal preservation of lumbar veins in the lower IVC stump is important when performing permanent interruption to ensure adequate collateral venous drainage [17]. Any areas of suspected caval wall invasion are sent for frozen section to ensure clear margins, as positive vascular margins confer a significantly increased risk of recurrence [74]. VTT directly invades the IVC wall in up to 23% of cases, most commonly at the renal vein ostium [75].

#### 5.4.4. Intraoperative Emergency Management

Despite meticulous technique, catastrophic intraoperative complications can occur and the surgical team must be prepared for immediate escalation. Tumor embolization during IVC manipulation has been reported in circa 1.5–3.4% of IVC thrombectomy cases [43,70], with a mortality rate of 60–75% when it occurs [70] (though this range derives from retrospective institutional reports and the true incidence may be higher in unselected populations). If acute respiratory distress develops from suspected intraoperative PE, prompt thoracotomy with pulmonary arteriotomy and clot extraction should be considered [69]. In the event of uncontrolled major hemorrhage in a patient not on bypass, temporary aortic cross-clamping above the celiac trunk may serve as a rescue maneuver; alternatively, emergent initiation of CPB should be pursued [69]. Fresh-frozen plasma, platelets, and packed red blood cells should be transfused liberally in hemorrhaging patients. These scenarios underscore the critical importance of cardiac surgery standby and immediate availability of bypass circuits for all Level II–III cases.

### 5.5. Cavorrhaphy and IVC Reconstruction

After the VTT is removed and the specimen is set aside, attention turns to repairing the IVC. The lumen is irrigated with heparinized saline and inspected—often with a gloved finger or flexible instrument—to ensure no residual tumor fragments or clots remain [5,69]. Prior to completing the closure, the patient is placed in Trendelenburg position and the anesthesia team is asked to apply positive airway pressure, while the distal (infrarenal) clamp is briefly released to allow back-bleeding through the open cava, flushing out any residual thrombus fragments, air, and debris from the IVC lumen [69]. Suction is used to aspirate blood and air, then the clamp is reapplied.

**Figure 10:**
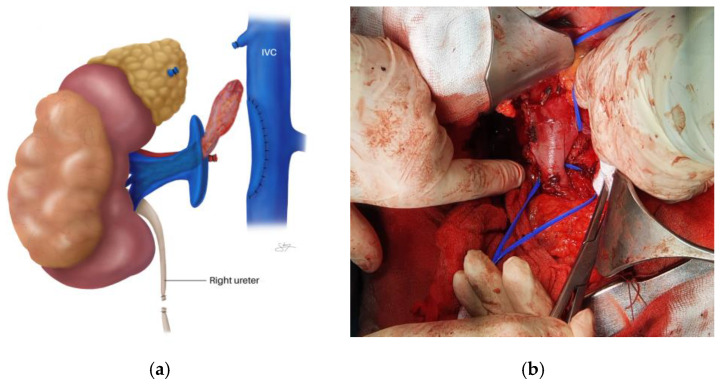
Side by side view of final cavorrhaphy after lateral cavotomy: (**a**) Illustration (by our graphic design expert Silvia Claudia Dobre). (**b**) Intraoperative image.

The cavotomy is closed primarily with a running polypropylene suture (typically 4-0 on a vascular needle), ensuring that the IVC lumen is not narrowed by more than approximately 50% of its preoperative caliber [69,70]. For a small lateral cavotomy, a single-layer running closure is sufficient and maintains excellent caval patency. Figure 10 provides a side-by-side comparison of the final cavorrhaphy after lateral cavotomy, with the illustrated schematic (Figure 10a) demonstrating the repair technique and the corresponding intraoperative image (Figure 10b) confirming the completed closure.

If the cavotomy was extensive (e.g., full anterior wall incision or a segment of caval wall excised) and vascular resection would narrow the IVC lumen by more than 50%, a patch graft repair can be performed to restore the IVC diameter. Autologous pericardium, bovine pericardium, or expanded polytetrafluoroethylene (ePTFE) can be sutured in to reconstruct the caval wall [5,69]. The patch should be slightly smaller than the cavotomy to maintain stretch of the repaired caval wall and minimize the risk of subsequent bland thrombus formation [69].

In cases of extensive IVC wall invasion by tumor, segmental resection of the IVC may be necessary. The ends of the IVC may be re-approximated primarily for short segments, while longer replacements may require an interposition graft; ePTFE is the preferred synthetic material given its low thrombogenic potential. IVC interruption with circumferential resection (by ligation, stapling, or oversewing) may be necessary when: (i) lesions involve more than half the circumference of the IVC; (ii) there is complete chronic obstruction without clinical symptoms of venous stasis; (iii) there is a high risk of postoperative PE due to unresectable bland thrombus; or (iv) successful thrombectomy has been complicated by vascular intima layer damage [17,70]. Importantly, IVC interruption can be accomplished below the level of the MHVs without major consequences if an adequate collateral venous network has been established [70]. In cases where the IVC is either ligated or permanently occluded, preservation of venous collaterals and lumbar veins not involved by tumor during IVC mobilization is critical to maintain venous return [5,17].

If Level III liver mobilization was performed, the hepatic ligaments are tacked back into place to prevent torsion of the liver [69]. Prior to tying the final knots of the IVC repair, air is evacuated to prevent embolism. Once the suture line is secured, the vascular clamps and tourniquets are removed in a controlled sequence: the suprarenal (proximal) clamp is released first, then the contralateral renal vein clamp, and lastly the infrarenal IVC clamp [5,53]. Restoration of venous flow is confirmed and the IVC repair is inspected for bleeding; if any bleeding point exists, additional stitches are placed. Close communication with anesthesiology is maintained during unclamping, as fluid shifts or residual tumor emboli can cause instability.

### 5.6. Specimen Evaluation and Final Operative Steps

**Figure 11:**
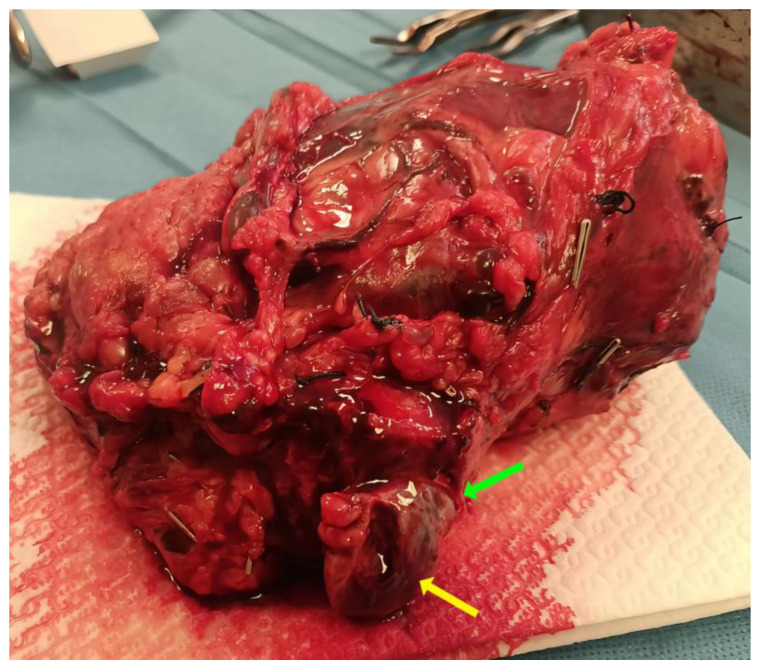
Nephrectomy specimen showing radically excised right-sided RCC, en bloc with Level I venous tumor thrombus. Yellow arrow indicates the tumor thrombus “cast” removed from the IVC, and the green arrow shows the bordering invaded renal vein, occupied in full by the thrombus.

At this stage, the kidney (if still attached by the renal vein) is freed and the specimen is removed. The final surgical specimen typically consists of the kidney, adrenal gland (if removed), any lymph node packet (if a lymphadenectomy was performed), and the entire VTT either within the excised renal vein or as a separate segment from the IVC. Figure 11 illustrates the end result: a nephrectomy specimen from a right-sided RCC with a Level I VTT, showing the entire VTT “cast” that was extracted from the IVC (yellow arrow) along with the involved renal vein segment occupied by the thrombus (green arrow).

The gross pathology is examined to confirm whether the IVC margin is clear of tumor. Frozen sections of the vascular margins may be obtained intraoperatively to verify complete resection, given the significant prognostic impact of margin status—positive vascular margins confer a significantly increased risk of recurrence [74].

Before closing the abdomen, the retroperitoneum is inspected for hemostasis and a closed suction drain is placed. If significant residual bland thrombus was identified during surgery that could not be fully evacuated, consideration may be given to perioperative anticoagulation, although this must be weighed against hemorrhagic risk. Hilar and/or regional lymphadenectomy may be performed for staging purposes. Postoperative monitoring in the intensive care unit is recommended for Level III cases [53,69].

Temporary hepatic dysfunction, characterized by elevated transaminases and alkaline phosphatase, is common after Level III procedures requiring suprahepatic IVC clamping and/or hepatic vascular exclusion; liver enzymes typically peak at 2–3 days postoperatively and gradually resolve [69]. This complication can be minimized by limiting porta hepatis clamping times and hepatic ischemia. Cardiac, renal, and intestinal ischemia can also occur during prolonged cross-clamping, particularly in patients with poor preoperative cardiac function, and postoperative creatinine monitoring is indicated for all patients undergoing IVC thrombectomy [69].

### 5.7. Thrombus Level–Specific Considerations: Synthesis

The general principles of vascular control, cavotomy, and cavorrhaphy described in the preceding sections are adapted in a stepwise fashion according to the thrombus level (Mayo classification). Table 1 provides an at-a-glance summary of the key operative parameters for each level, including the extent of IVC dissection, requirement for liver mobilization, method of vascular control, cavotomy approach, and need for circulatory support. It is important to recognize that although these levels form a useful conceptual framework, individual thrombus anatomy—including the degree of IVC occlusion, presence of bland thrombus, caval wall invasion, and development of collateral venous pathways—may necessitate intraoperative deviation from the standard algorithm. Intraoperative TEE and close communication between the surgical and anesthesia teams remain the cornerstone of adaptive decision-making during these complex procedures.

As summarized in Table 1, the operative complexity increases in a stepwise fashion from Level 0 to Level III. The most consequential transitions occur between Level I and Level II—where full IVC cross-clamping and lumbar vein ligation become necessary—and between Level II and Level III—where liver mobilization and hepatic vascular exclusion enter the operative plan. The incidence data highlight that the majority of VTT cases (~77%, Levels 0–I combined) can be managed with relatively straightforward techniques, while the minority requiring extensive hepatic mobilization (~19%, Levels II–III combined) represent the most technically complex cases [4,69].

All in all, open surgical resection remains the gold standard for RCC with infradiaphragmatic VTT. By tailoring the surgical approach to the thrombus level—ranging from simple control of the renal vein for Level 0 to full liver mobilization and hepatic vascular exclusion for Level III—surgeons can achieve en bloc removal of the kidney and VTT in the vast majority of cases without CPB. These operations demand detailed preoperative planning and a multidisciplinary team, but when executed successfully, they offer the patient the best chance for long-term cure. The techniques described above, complemented by intraoperative imaging (particularly TEE) and refined surgical instruments, form the foundation of contemporary management for RCC with Levels 0–III VTT.

## 6. Postoperative Outcomes and Oncological Management

Long-term survival following surgical management of RCC with VTT has improved substantially due to refined surgical techniques and the addition of multimodal therapy. A Mayo Clinic series of 540 patients reported 5-year cancer-specific survival rates of 49.1% for VTT confined to the renal vein (level 0), and 31.7%, 26.3%, 39.4%, and 37.0% for IVC thrombi at levels I, II, III, and IV respectively [4]. Notably, while patients with any IVC involvement had worse outcomes than those with renal-vein-only thrombus (*p* = 0.002), no significant survival differences were seen between the individual IVC dissemination levels I–IV (*p* = 0.868), emphasizing that VTT level influences surgical complexity more than prognosis [4,21]. Contemporary series show further improved outcomes with modern care: specifically, one-year overall survival now approaches 90% for patients with localized disease [76], with 2-year overall survival around 75–80% for RCC patients with VTT [77,78], and 5-year survival rates of approximately 50–60% can be achieved in non-metastatic cases [4,5,78]. These advances underscore that aggressive surgical management, when feasible, can markedly alter the natural history of RCC with VTT compared to the dismal prognosis of untreated disease (historically a median survival of only ~5 months) [3].

It is important to contextualize these figures historically. The widely cited Mayo Clinic level-specific survival data [4] derive from a cohort treated between 1970 and 2000, predating both modern perioperative protocols and adjuvant immunotherapy. The reported 5-year cancer-specific survival rates of 26–49% across VTT levels reflect an era without routine intraoperative TEE, contemporary blood conservation strategies, or postoperative immune checkpoint inhibitors. Contemporary series from 2020–2025, though generally lacking equivalent sample sizes and follow-up duration, suggest meaningfully improved outcomes: one-year overall survival now approaches approximately 90% for patients with localized disease [76], two-year overall survival approximately 75–80% [77,78], and five-year cancer-specific survival of approximately 50–60% in non-metastatic cases [4,5,78]. Direct comparison across eras is limited by differences in patient selection criteria, surgical technique, perioperative care, availability of adjuvant systemic therapy, and reporting standards. Prospective, multi-institutional registries with standardized follow-up are needed to provide more robust contemporary benchmarks.

Multiple clinicopathologic factors independently predict oncological outcomes beyond just VTT level. The presence of metastatic disease at diagnosis confers the poorest prognosis (e.g., AJCC stage IV disease carries a hazard ratio [HR] ~2.85 for worse survival) [21]. Similarly, regional lymph node involvement and sarcomatoid tumor differentiation are associated with significantly higher risks of disease progression and cancer-specific mortality. Surgical margin status is critical—incomplete resection or positive margins roughly double the risk of cancer-related death (HR ~2.54) [21].

IVC wall invasion, which should be suspected on imaging when the IVC diameter exceeds 25 mm, necessitates more extensive resection (often including a segment of the IVC wall). Nevertheless, if the involved vessel wall is completely excised and reconstruction is successful, survival outcomes are comparable to those without vascular parietal invasion (yet with complete tumor excision), reinforcing the importance of achieving R0 on final pathology [74]. Tumor histology also influences outcomes: clear-cell RCC tends to have superior cancer-specific survival compared to papillary and sarcomatoid variants (HR 1.62 for papillary vs. clear cell), highlighting the more aggressive biology of non-clear-cell and sarcomatoid disease variants [79].

### 6.1. Adjuvant Systemic Therapy

Adjuvant systemic immunotherapy has recently transformed the postoperative landscape of oncological management for locally advanced RCCs with VTT [80,81]. Historically, cytokine therapies or TKIs given after nephrectomy did not conclusively improve survival in high-risk RCC (e.g., the ASSURE trial was negative for adjuvant sunitinib/sorafenib [82]). Table 2 summarizes the landmark trials informing perioperative systemic therapy. Among these, only KEYNOTE-564 demonstrated a significant benefit: one year of adjuvant pembrolizumab (anti–PD-1, 200 mg every 3 weeks for 17 cycles) reduced the risk of recurrence or death by approximately 32% (HR 0.68, *p* ≈ 0.002), with a confirmed overall survival benefit at 4 years (91.2% vs. 86.0%) [80,81]. In contrast, CheckMate 914 (adjuvant nivolumab + ipilimumab for 6 months) did not significantly improve DFS [83], and the PROSPER trial found no benefit for perioperative nivolumab monotherapy versus surgery alone [84]. Thus, pembrolizumab monotherapy currently holds the highest level of evidence in the adjuvant setting [33,34].

On the basis of this, adjuvant pembrolizumab is currently approved and recommended by clinical guidelines for patients with high-risk features after nephrectomy, which includes virtually all RCCs with VTT (any pT3 tumor being high-risk by definition) [33,34]. Thereby, adjuvant immunotherapy is an important consideration following successful surgery. In practice, following radical surgery, RCC patients are generally referred to medical oncology once they have adequately healed (typically 4–12 weeks post-op) to discuss further adjuvant therapy. Generally, if the patient has clear-cell histology, no contraindication to immunotherapy, and high-risk traits (pT ≥ 3, Fuhrman grade ≥ 2), postoperative oncological management must strongly consider one year of pembrolizumab given the significant possibility of recurrence. Alternatively, for non-clear-cell tumors or in those who cannot tolerate immunotherapy, close surveillance may be employed instead [33,34].

### 6.2. Metastatic Disease Management

Approximately 20–30% of patients with RCC and an IVC thrombus present with synchronous distant metastases at diagnosis [5,21]. In this metastatic (M1) setting, management must be highly individualized, with systemic therapy serving as the cornerstone of treatment (typically combined ICIs or a TKI + ICI regimen). However, carefully selected M1 RCC patients may derive benefit from cytoreductive nephrectomy and thrombectomy, even in the presence of metastases [53]. Surgical intervention tends to be favored in scenarios such as when the metastatic burden is limited and amenable to local treatment (oligometastatic disease), the patient has a good performance status, and/or the primary tumor/thrombus are causing significant symptoms and/or poses acute complication risks (for example, threat of PE from IVC obstruction or Budd-Chiari syndrome due to hepatic vein involvement). In such cases, even though the presence of metastases markedly worsens prognosis, aggressive surgical management can yield significant symptom palliation and may even potentially prolong survival, especially now that effective systemic therapies can address micro-metastatic and/or residual disease [5,53]. Even so, if the metastatic burden is high and/or the patient’s overall condition is poor, the risks and morbidity of an extensive operation often outweigh the benefit, and thus upfront surgery is usually avoided in favor of systemic therapy alone [34].

Treatment sequencing remains an important consideration in metastatic disease. The CARMENA trial established that omitting upfront cytoreductive nephrectomy in favor of sunitinib alone does not compromise survival [85], while the SURTIME trial suggested a possible overall survival advantage to deferred surgery (median OS 32.4 vs. 15.0 months; HR 0.57), although the primary endpoint (28-week progression-free rate) was not met [86] (Table 2). The PROSPER trial’s negative results for perioperative nivolumab monotherapy further underscore that the role of perioperative immunotherapy in this population remains undefined [84]. These findings collectively support a selective, response-guided approach to cytoreductive surgery in the metastatic setting, with definitive recommendations awaiting the results of ongoing randomized trials.

When surgery is undertaken in a metastatic RCC with VTT, a comprehensive approach to tumor reduction is often pursued. Surgeons may attempt to resect not only the kidney and thrombus but also any accessible metastatic lesions—for example, a solitary lung nodule or adrenal metastasis—either concurrently during the same operation or in a planned staged procedure shortly thereafter [5,53]. This aggressive local therapy aims for maximal tumor clearance, which in turn may prolong survival or provide better palliation of symptoms in the metastatic setting. Ultimately, the management of metastatic RCC with VTT requires a multidisciplinary discussion on a case-by-case basis [5,53]. The surgical risks and complexities must be weighed against the potential oncological benefit, with input from urology, medical oncology, and other specialties to formulate the optimal plan for each individual patient [33,34].

Postoperatively, patients are closely monitored. An initial imaging (MRI) at 1–2 weeks post-op is advocated by some to ensure no residual IVC thrombus [5]. Follow-up thereafter includes periodic chest and abdominal imaging, as these patients have significant relapse risks (locally/distantly). If a recurrence is detected confined to the IVC, surgical re-intervention can be considered in select cases, though this is rare [53]. Key prognostic determinants—including metastatic status at diagnosis, regional lymph node involvement, sarcomatoid or non-clear-cell histology, tumor grade, and surgical margin status—have already been discussed above. Beyond these, primary tumor size > 7 cm, perinephric or renal sinus fat invasion, and direct IVC wall invasion (pT3c) are associated with higher recurrence risk [74]. Distant metastases to the lung, bone, and liver represent the predominant mode of failure, whereas local IVC-site recurrence is rare in contemporary series when complete resection with negative vascular margins is achieved [5,53].

## 7. Emerging Minimally Invasive Approaches

Open surgery has long been the standard for RCC with VTT, but minimally invasive approaches—laparoscopic or robot-assisted thrombectomy—have rapidly evolved in the last two decades. The first laparoscopic IVC thrombectomy in RCC was reported around 2000 [87], and the first robotic-assisted case series appeared in 2011 [7]. Since then, numerous small series have demonstrated the feasibility of managing select tumor thrombi laparoscopically or robotically [88,89]. Minimally invasive surgery offers potential benefits of reduced blood loss, shorter hospital stays, and quicker recovery, but applying it to IVC thrombectomy is highly challenging. The complexity of controlling a major vein and extracting a large thrombus through small ports means these techniques demand significant expertise and often longer operative time. They are generally considered only for lower-level thrombi (level I–II), although a few centers have reported robotic thrombectomy even for level III cases with skilled teams [90,91].

Patient selection remains critical: right-sided tumors, limited comorbidities, and absence of IVC wall invasion identify optimal candidates [92,93]. Robotic-assisted laparoscopy has largely supplanted pure laparoscopy for all but the simplest cases, offering superior dexterity, tremor filtration, and wristed instrument control that facilitate intracorporeal vascular dissection and suturing [92]. The fundamental operative steps mirror the open approach—early arterial control, circumferential IVC dissection, vascular clamping, cavotomy, thrombus extraction, and cavorrhaphy—adapted for the intracorporeal environment with laparoscopic bulldog clamps or tourniquets for vascular control [92,94]. A transperitoneal approach is generally preferred for the additional working space it provides [93,94]. For level III cases performed robotically, full liver mobilization and a Pringle maneuver have been described, though such cases remain confined to highly experienced centers [90,95].

Importantly, minimally invasive thrombectomy should reproduce the oncologic quality of open surgery—i.e., en bloc tumor removal without spillage. Case series suggest that oncologic outcomes (margin status, recurrence patterns) are similar between robotic and open approaches in experienced hands [96,97]. A recent meta-analysis comparing robotic vs. open IVC thrombectomy found no significant difference in positive margin rates or survival, but the robotic approach was associated with less blood loss, fewer transfusions, shorter hospital stay, and fewer complications overall [93]. Operative times in robotics have come down as surgeons gain experience, and can be comparable to open in some centers [98]. Recent series report conversion rates of <5% to open surgery, with median operative times of 240–350 min and estimated blood loss of 160–240 mL for appropriately selected cases [92,93].

However, these favorable results must be interpreted with caution: almost all published series come from high-volume tertiary centers with inherent patient selection bias favoring smaller, non-invasive thrombi. Additionally, the absence of standardized outcome reporting (variable definitions of blood loss, complication grading, and follow-up duration) across robotic series makes quantitative comparison with open benchmarks unreliable. Publication bias likely further inflates the apparent advantages of the robotic approach, as unfavorable outcomes and conversions may be underreported.

Technical limitations include the inability to manually palpate structures and the difficulty of extracting bulky thrombi through ports without fragmentation. The learning curve is steep, with expert proficiency estimated to require 80–120 procedures [98]. Standardization of technique remains lacking, and current guidelines have not endorsed minimally invasive thrombectomy as a new standard, reserving it for centers with appropriate expertise [33]. The general consensus is that open surgery remains standard for Level III thrombi, while robotic approaches may be considered for Level I–II in selected patients at experienced centers, with the understanding that conversion to open surgery may be necessary [33,93].

In summary, robotic-assisted thrombectomy is technically feasible and shows favorable perioperative outcomes for Level I–II thrombi at experienced centers, but long-term oncological data and prospective comparative trials against the open approach remain lacking. Open surgery therefore remains the reference standard, particularly for Level III thrombi and cases with IVC wall invasion. The choice of approach should be governed by thrombus anatomy, institutional expertise, and the overriding priority of complete, safe en bloc tumor removal.

## 8. Conclusions

RCC with VTT extending into the infradiaphragmatic IVC remains among the most complex challenges in urologic oncology, demanding a synthesis of oncological principles, advanced vascular surgical techniques, and coordinated multidisciplinary expertise. Open radical nephrectomy with en bloc IVC thrombectomy continues to represent the gold standard of treatment, offering the only realistic path toward long-term cure in patients with non-metastatic disease. The operative approach must be precisely tailored to the VTT level: from simple ostial excision and partial IVC clamping for Level 0–I thrombi, through extensive circumferential IVC dissection for Level II disease, to full piggyback liver mobilization with hepatic vascular exclusion for Level III thrombi. Meticulous preoperative imaging—combining multiphase CT, MRI, and intraoperative TEE—is indispensable for accurate VTT classification, assessment of IVC wall invasion, and adaptive intraoperative decision-making. Equally critical are thorough patient optimization, including frailty assessment, nutritional screening, and coordination of blood product availability, as well as centralization of care at high-volume institutions where multidisciplinary surgical teams can deliver these procedures with perioperative mortality rates < 2%.

The therapeutic landscape is rapidly evolving. Adjuvant pembrolizumab has established a new standard of postoperative care for high-risk patients following the KEYNOTE-564 trial, while neoadjuvant combination regimens of ICIs with TKIs show considerable promise in VTT downstaging, potentially reducing surgical complexity and morbidity—albeit without demonstrated survival benefit to date. Robotic-assisted thrombectomy is emerging as a viable alternative for appropriately selected Level I–II cases at centers with adequate expertise, with meta-analytic data suggesting comparable oncological outcomes alongside reduced blood loss and shorter hospital stays [93], though prospective comparative trials remain lacking. However, open surgery remains the reference standard for Level III thrombi and for cases involving IVC wall invasion or extensive bland thrombus. Ultimately, the management of RCC with VTT exemplifies the principle that surgical self-reliance—grounded in detailed anatomical knowledge, rigorous preoperative preparation, and the ability to escalate intraoperatively when needed—remains the cornerstone upon which favorable patient outcomes are built. Continued investigation into neoadjuvant strategies, biomarker-guided patient selection, and the expanding role of minimally invasive techniques will further refine and improve the care of this challenging RCC patient population.

## Figures and Tables

**Table 1:** Summary of Thrombus Level-Specific Operative Considerations (Mayo Levels 0–III).

Feature	Level 0	Level I	Level II	Level III
Definition	Confined to the RV.	Into the IVC, but ≤2 cm ↑ the RV ostium.	>2 cm ↑ the RV, but ↓ the MHVs.	↑ the MHVs, but ↓ the diaphragm.
Incidence (%, RCCs/VTTs)	12%/65%	2%/12%	3%/14%	1%/5%
IVC dissection	Minimal: ↑ + ↓ the RV.	Limited: infrarenal + short suprarenal seg.	Extensive: infrarenal to infrahepatic IVC (with LV ligation).	Complete: infrarenal to suprahepatic IVC + retrohepatic seg.
Liver mobilization	NR	NR	Usually NR; division of SHVs may be helpful as VTT approaches the MHVs (gains 2–5 cm).	Full “piggyback” required: division of all hepatic ligaments + SHVs to expose retro/suprahepatic IVC.
Vascular control	Satinsky-Cs just ↑ the RV ostium (with partial IVC occlusion).	Rummel-Ts or Satinsky-Cs, with full IVC cross-C. (if VTT mobile or imaging not recent).	Sequential Rummel-Ts: infrarenal IVC → contralateral RV → suprarenal IVC (↑VTT extent).	Sequential Rummel-Ts (Level II) + Pringle maneuver (HVE) if VTT cannot be “milked” ↓ MHVs; consider transdiaphragmatic intrapericardial IVC control.
Test clamp	NR	Recommended if full IVC cross-C. is performed.	Mandatory	Mandatory; CO drop >50% or MAP drop > 30% → bypass.
Cavotomy (CVT) approach	Circ. excision of RV ostium; no formal CVT.	“Milking” into RV + ostial excision; limited lateral CVT if VTT too bulky.	Lateral, midline, or “L”-shaped CVT; lateral preferred if VTT can be “milked” toward ostium.	Lateral or midline CVT; 2-step CVT if VTT cannot be “milked” ↓ MHVs.
Circulatory support (bypass)	NR	NR	Generally NR → collateral return via lumbar–azygos and portal systems is usually adequate.	Usually NR (in experienced centers); VVB if hemodynamically unstable; CPB/DHCA reserved for uncontrollable instability.
Pringle maneuver	No	No	No (unless caudate SHV division requires hepatic inflow control).	Yes, if suprahepatic IVC clamping ↑ MHVs is required (max. 60 min; ≤20 min preferred).
Intraop. TEE	Optional	Optional	Recommended	Mandatory
Key technical pearls	“Milk” VTT into RV; Satinsky-C. partially occludes IVC preserving flow; gauze wrap on RV stump prevents tumor spillage.	Rummel-Ts preferred over clamps (less likely to fracture VTT); lateral clamping with 2 Satinsky traps VTT during venotomy; must back-bleed before completing CVR.	Ligation of all LVs for circ. IVC control; “L”-shaped CVT over RV ostium; caudate SHV division gains cephalad exposure; contralateral RA clamping may prevent engorgement (especially in left-sided tumors).	“Piggyback” liver mobilization (transplant technique) = key enabling maneuver; two-step CVT + temporary HVE preserves hepatic venous bypass; team must be prepared for rapid escalation (VVB/CPB).

Abbreviations: RV = renal vein; IVC = inferior vena cava; intraop. = intraoperative; VTT = venous tumor thrombus; MHVs = major hepatic veins; SHVs = accessory caudate lobe short hepatic veins; LV(s) = lumbar vein(s); Satinsky-C(s) = Satinsky clamp(s); Rummel-Ts = Rummel tourniquets; cross-C. = cross-clamping; CVT = cavotomy; CVR = cavorrhaphy; Circ. = circumferential; HVE = hepatic vascular exclusion; CO = cardiac output; MAP = mean arterial pressure; TEE = transesophageal echocardiography; VVB = veno-venous bypass; CPB = cardiopulmonary bypass; DHCA = deep hypothermic circulatory arrest; RA = renal artery; NR = not required; seg. = segment; ↑ = above/cephalad to; ↓ = below/caudal to.

**Table 2:** Summary of landmark clinical trials informing perioperative systemic therapy for RCC with VTT.

Trial	Phase/Design	Setting	Regimen	Key Endpoint	Result
**KEYNOTE-564** [80,81]	Phase III, RCT, double-blind	Adjuvant (post-Nx, high-risk)	Pembrolizumab 200 mg q3w × 17 cyc vs. placebo	DFS (primary); OS (secondary)	DFS: HR 0.68 (*p* ≈ 0.002); 2-yr DFS 77% vs. 68%; 4-yr OS 91.2% vs. 86.0%
**CheckMate 914** [83]	Phase III, RCT, double-blind	Adjuvant (post-Nx, localized)	Nivolumab + ipilimumab (6 mo) vs. placebo	DFS (primary)	DFS not significantly improved vs. placebo
**PROSPER (EA8143)** [84]	Phase III, RCT, open-label	Perioperative (pre + post-Nx, high-risk)	Nivolumab mono (periop) vs. observation	RFS (primary)	No benefit vs. surgery + surveillance alone
**CARMENA** [85]	Phase III, RCT	Metastatic (1st-line)	Sunitinib alone vs. CN + sunitinib	OS (primary)	No survival disadvantage to omitting CN (non-inferiority met)
**SURTIME** [86]	Phase III, RCT	Metastatic (deferred vs. immediate CN)	Sunitinib × 3 cyc → CN → sunitinib vs. immediate CN → sunitinib	28-wk PFS (primary); OS (secondary)	28-wk PFS: no improvement; mOS: 32.4 vs. 15.0 mo (HR 0.57, exploratory)
**ASSURE (E2805)** [82]	Phase III, RCT, double-blind	Adjuvant (post-Nx, high-risk)	Sunitinib or sorafenib vs. placebo	DFS (primary)	Negative—no DFS improvement

Abbreviations: CN = cytoreductive nephrectomy; cyc = cycles; DFS = disease-free survival; HR = hazard ratio; mo = months; mOS = median overall survival; Nx = nephrectomy; OS = overall survival; PFS = progression-free survival; q3w = every 3 weeks; RCT = randomized controlled trial; RFS = recurrence-free survival; wk = week.

## Data Availability

Data available on request from the corresponding authors.

## Abbreviations

The following abbreviations are used in this manuscript:

3D	Three-Dimensional
AJCC	American Joint Committee on Cancer
ASA	American Society of Anesthesiologists
AUC	Area Under the Curve
CEUS	Contrast-Enhanced Ultrasound
CI	Confidence Interval
CO	Cardiac Output
CPB	Cardiopulmonary Bypass
CPET	Cardiopulmonary Exercise Testing
CT	Computed Tomography
DFS	Disease-Free Survival
DHCA	Deep Hypothermic Circulatory Arrest
EAU	European Association of Urology
ECOG	Eastern Cooperative Oncology Group
ePTFE	Expanded Polytetrafluoroethylene
18F-FDG	18F-Fluorodeoxyglucose
HR	Hazard Ratio
HVE	Hepatic Vascular Exclusion
ICI	Immune Checkpoint Inhibitor
ICU	Intensive Care Unit
INR	International Normalized Ratio
IO	Immuno-Oncology
IOCS	Intraoperative Cell Salvage
IVC	Inferior Vena Cava
LMWH	Low Molecular Weight Heparin
MAP	Mean Arterial Pressure
MHVs	Major Hepatic Veins
MRI	Magnetic Resonance Imaging
NCCN	National Comprehensive Cancer Network
OR	Odds Ratio
pCR	Pathological Complete Response
PD-1	Programmed Death-1
PE	Pulmonary Embolism
PET/CT	Positron Emission Tomography/Computed Tomography
PNI	Prognostic Nutritional Index
PSMA	Prostate-Specific Membrane Antigen
RCC	Renal Cell Carcinoma
SHVs	Short Hepatic Veins (Accessory Caudate Lobe Veins)
SUVmax	Maximum Standardized Uptake Value
TEE	Transesophageal Echocardiography
TKI	Tyrosine Kinase Inhibitor
VEGF	Vascular Endothelial Growth Factor
VO2	Oxygen Consumption
VTE	Venous Thromboembolism
VTT	Venous Tumor Thrombus
VVB	Veno-Venous Bypass
